# Supplementary material for: Multidimensional chromatin profiling of zebrafish pancreas to uncover and investigate disease-relevant enhancers
Source: Nat Commun. 2022 Apr 11;13:1945. doi: 10.1038/s41467-022-29551-7 (PMC9001708; doi:10.1038/s41467-022-29551-7)
Supplement: Supplementary file 2 — Supplementary figures1-10 [file 41467_2022_29551_MOESM2_ESM.pdf]

Supplementary figure 1

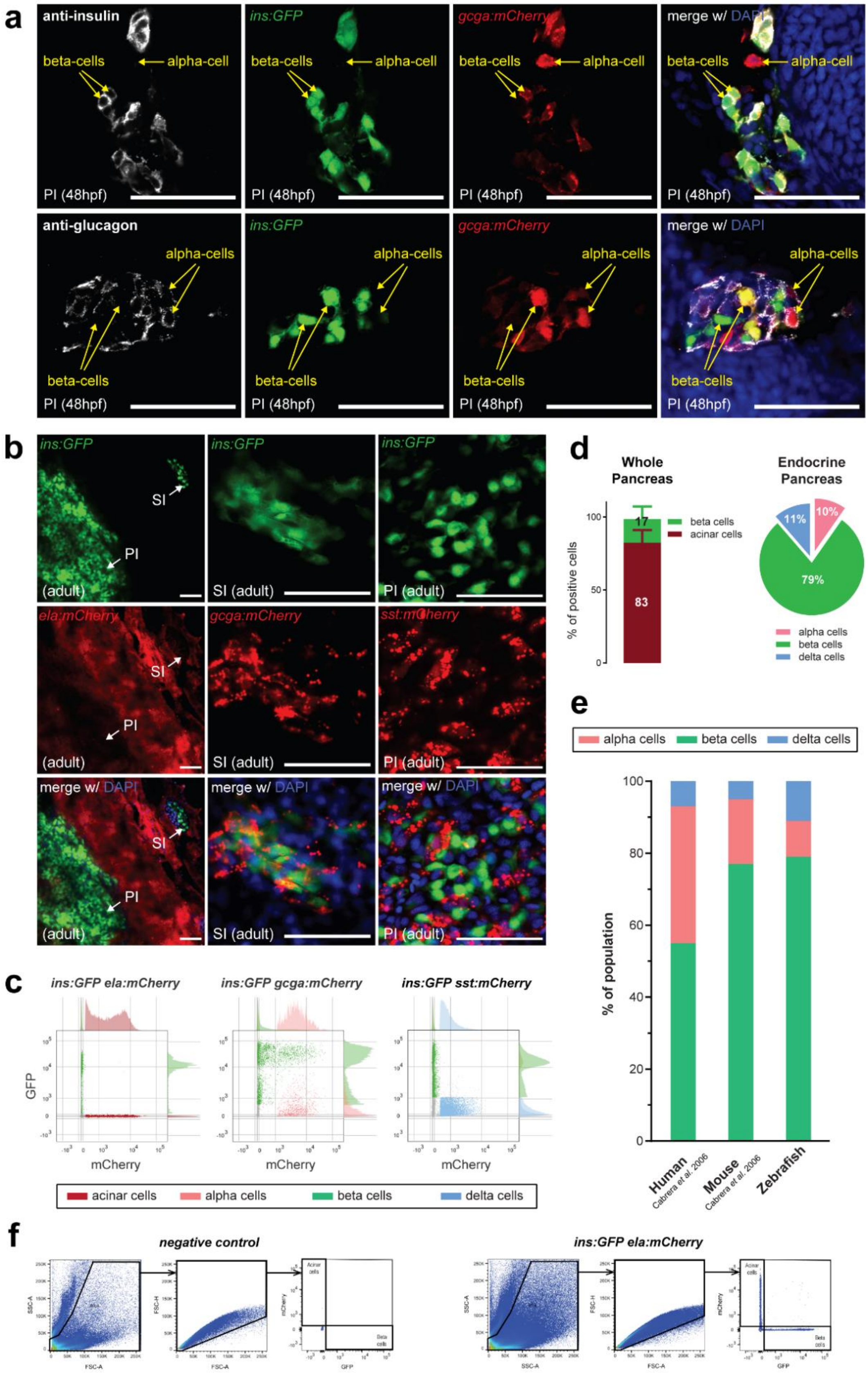

Supplementary Fig. 1. Basic constitution of the zebrafish adult pancreas. a Representative confocal images of the principal islet (PI) from 48hpf Tg(*ins:GFP, gcga:mCherry*) zebrafish embryos stained with antibodies directed against insulin (white, top panels, n=3) or glucagon (white, bottom panels, n=8). The GFP reporter (green) is expressed exclusively in beta cells (defined by insulin immunostaining), while the mCherry reporter (red) is expressed in alpha cells (defined by glucagon immunostaining) as well as a subset of the beta-cell population. Arrows show the location of some insulin-producing beta cells and glucagon-producing alpha cells. Nuclei are stained with DAPI. Scale bar: 50 µm. **b** Representative confocal images of whole-mounted pancreatic tissue from adult Tg(*ins:GFP*, *ela:mCherry*) (left panels, n=4), Tg(*ins:GFP*, *gcga:mCherry*) (middle panels, n=2), and Tg(*ins:GFP*, *sst:mCherry*) zebrafish (right panels, n=1). The adult endocrine pancreas consists of a larger principal islet (PI) and smaller secondary islets (SI) embedded within the pancreatic exocrine tissue composed of acinar cells (red) and a network of duct cells. The PI and SIs are composed of the three major cell populations of beta cells, alpha cells and the somatostatin-producing delta cells, among others. Nuclei are stained with DAPI. Scale bar: 50 µm. **c** Representative scatter plots of flow cytometry analysis of adult Tg(*ins:GFP*, *ela:mCherry*), Tg(*ins:GFP*, *gcga:mCherry*), and Tg(*ins:GFP*, *sst:mCherry*) zebrafish pancreas. **d** Left panel: Relative percentage of pancreatic endocrine beta cells and exocrine acinar cells (mean ± SD) quantified by flow cytometry from adult Tg(*ins:GFP*, *ela:mCherry*) zebrafish pancreas (n=5). From this quantification we propose that the zebrafish exocrine pancreas is around 5-fold more abundant than the endocrine pancreas. The overrepresentation of the exocrine compartment of the pancreas compared to the endocrine compartment is also observed in the mammalian pancreas with 1-2% of the mouse adult pancreas being made up of beta cells[39] and human islets occupying between 1-3% of the total adult pancreatic mass[39-41]. Right panel: Relative percentage of pancreatic endocrine beta cells, alpha cells and delta cells, quantified by flow cytometry from adult Tg(*ins:GFP*, *gcga:mCherry*) (n=65) and Tg(*ins:GFP*, *sst:mCherry*) pancreas (n=30). **e** Comparison of the relative cell composition of the adult zebrafish endocrine pancreas with that of human and mouse islets. In the three species, the endocrine pancreas is composed mainly of beta-cells, followed by alpha cells and delta cells. Abbreviations: ela, elastase; gcga, glucagon; ins, insulin; sst, somatostatin. **f** Representative plots for adult wild-type zebrafish (negative control, left panels) and adult Tg(ins:GFP, ela:mCherry) whole pancreas (right panels) illustrating the gating strategy for flow cytomety analysis: FSC/SSC gate to identify living

cells, FSC-H/FSC-A to identify single cells types, and single cells types are gated according with positivity/negativity for reporter expression (representative plots can be found in c): in Tg(ins:GFP, ela:mCherry) animals, the beta-cell population is defined by gating ins:GFP positive ela:mCherry negative single cells, and the acinar cell population is defined by gating ins:GFP negative ela:mCherry positive single cells; in Tg(ins:GFP, gcga:mCherry) animals, the beta-cell population is defined by gating ins:GFP positive single cells, and the alpha-cell population is defined by gating ins:GFP negative gcga:mCherry positive single cells; in Tg(ins:GFP, sst:mCherry) animals, the beta-cell population is defined by gating ins:GFP positive sst:mCherry negative single cells, and the delta-cell population was defined by gating ins:GFP negative sst:mCherry positive single cells. Data included in Source Data file for **d**, and **e**.

Supplementary figure 2

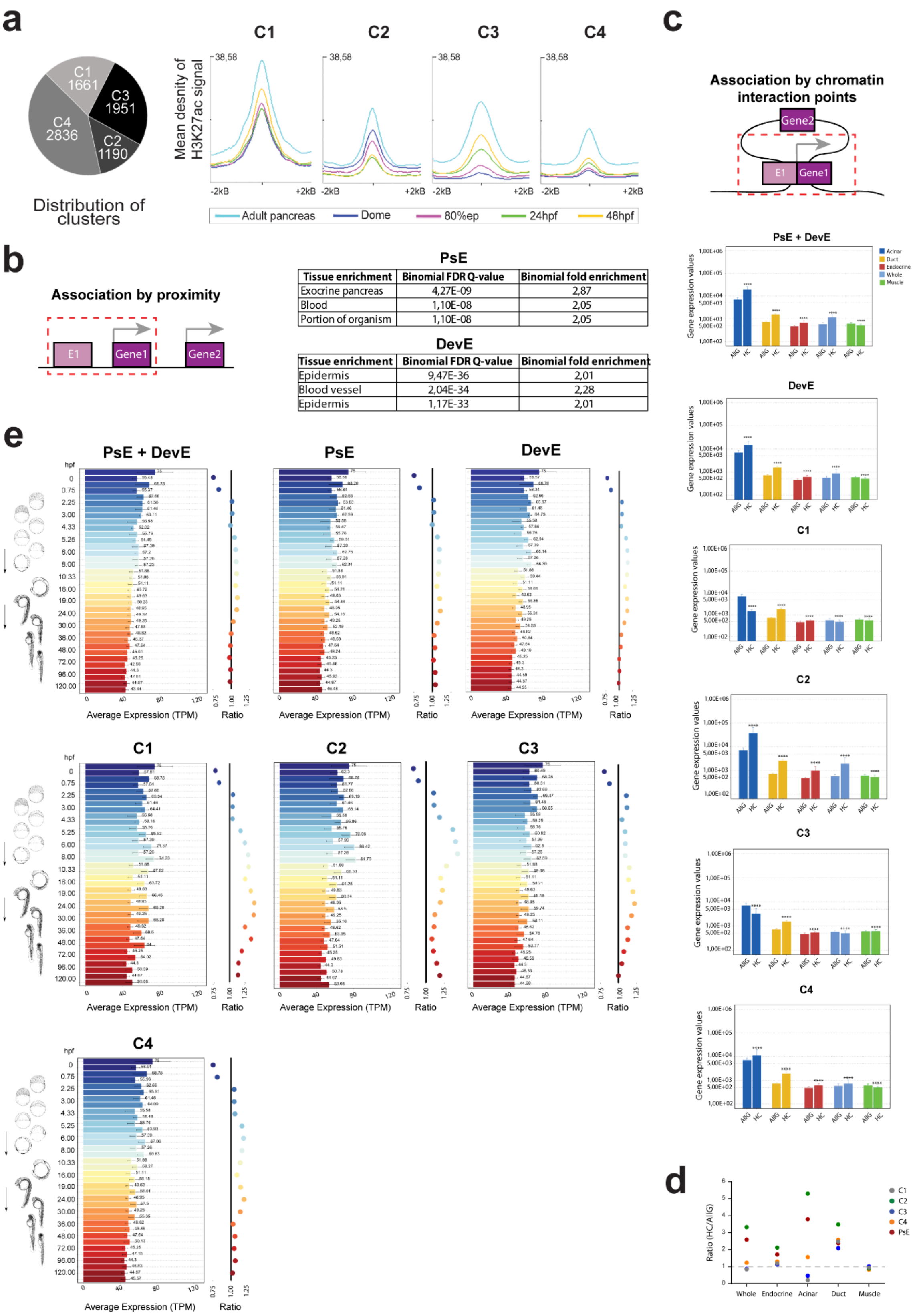

PsE

Tissue enrichment	Binomial FDR Q-value	Binomial fold enrichment
Exocrine pancreas	4,27E-09	2,87
Blood	1,10E-08	2,05
Portion of organism	1,10E-08	2,05

DevE

Tissue enrichment	Binomial FDR Q-value	Binomial fold enrichment
Epidermis	9,47E-36	2,01
Blood vessel	2,04E-34	2,28
Epidermis	1,17E-33	2,01

Supplementary Fig. 2. Average gene expression of the predicted target genes of different clusters of pancreatic enhancers. a Left Panel: number of sequences contained in each of the four clusters observed in DevE: C1, Cluster 1; C2, Cluster 2; C3, Cluster 3; and C4, Cluster 4. Right Panels: mean density of H3K27Ac signal for each cluster, centered in its summit and expanded ±2kb. ChIP-seq data for H3K27ac obtained from adult pancreas and embryos at different developmental stages (dome, 80% epiboly, 24hpf and 48hpf). **b** Left panel: Schematic representation of gene-to-enhancer association by genomic proximity with GREAT[45]. Right Panels: Tissue specific expression enrichment for genes associated to PsE and PsE+DevE. **c** Upper Panel: Schematic representation of gene-to-enhancer association by chromatin interaction points defined by HiChIP for H3K4me3 (HC). Lower Panels: Average expression of genes interacting with different enhancer sets detected by HC in adult zebrafish pancreas (PsE+DevE,n=8840 genes, DevE, n=5449 genes, C1, n=1917 genes, C2, n=1402 genes, C3, n=1888 genes and C4, n=2531 genes), compared to the average expression of all genes (AllG, n=33737 genes). Gene expression was determined by RNA-seq from pancreatic cells (acinar n=4; duct n=3; endocrine n=4), whole pancreas (n=2) and muscle (control, n=2). One-sided Wilcoxon tests (≥), *p*-values<0.05 considered significant. PsE+DevE,*****p*<2E-16; DevE and C1-C4, *****p*<0.0001. Error bars represent the 95% confidence interval. **d** Ratio between the average expression of genes interacting with pancreas-specific enhancers (PsE, C1, C2, C3 and C4 clusters; HC) and the average expression of all genes throughout different pancreatic zebrafish tissues (exocrine, endocrine, acinar, duct; AllG). The muscle was used as control. **e** At the left side of each panel, the average gene expression (transcripts per million, TPM) detected from RNA-seq from zebrafish embryos at different developmental stages (0 to 120hpf) is plotted. The top bar of each color is the average expression of genes associated with pancreas

enhancers (PsE+DevE,n=8840 genes, DevE, n=5449 genes, C1, n=1917 genes, C2, n=1402 genes, C3, n=1888 genes and C4, n=2531 genes) and the bottom bar of each color is the average expression of all genes (AllG, n=33737 genes), with the respective value depicted for each bar. On the right side of each panel is depicted the ratio between the average expression of all genes (HC/AllG) and the average expression of genes associated with pancreas enhancers, maintaining the same color code. Error bars represent the 95% confidence interval. Data included in Source Data file for **a**-**e**.

Supplementary figure 3

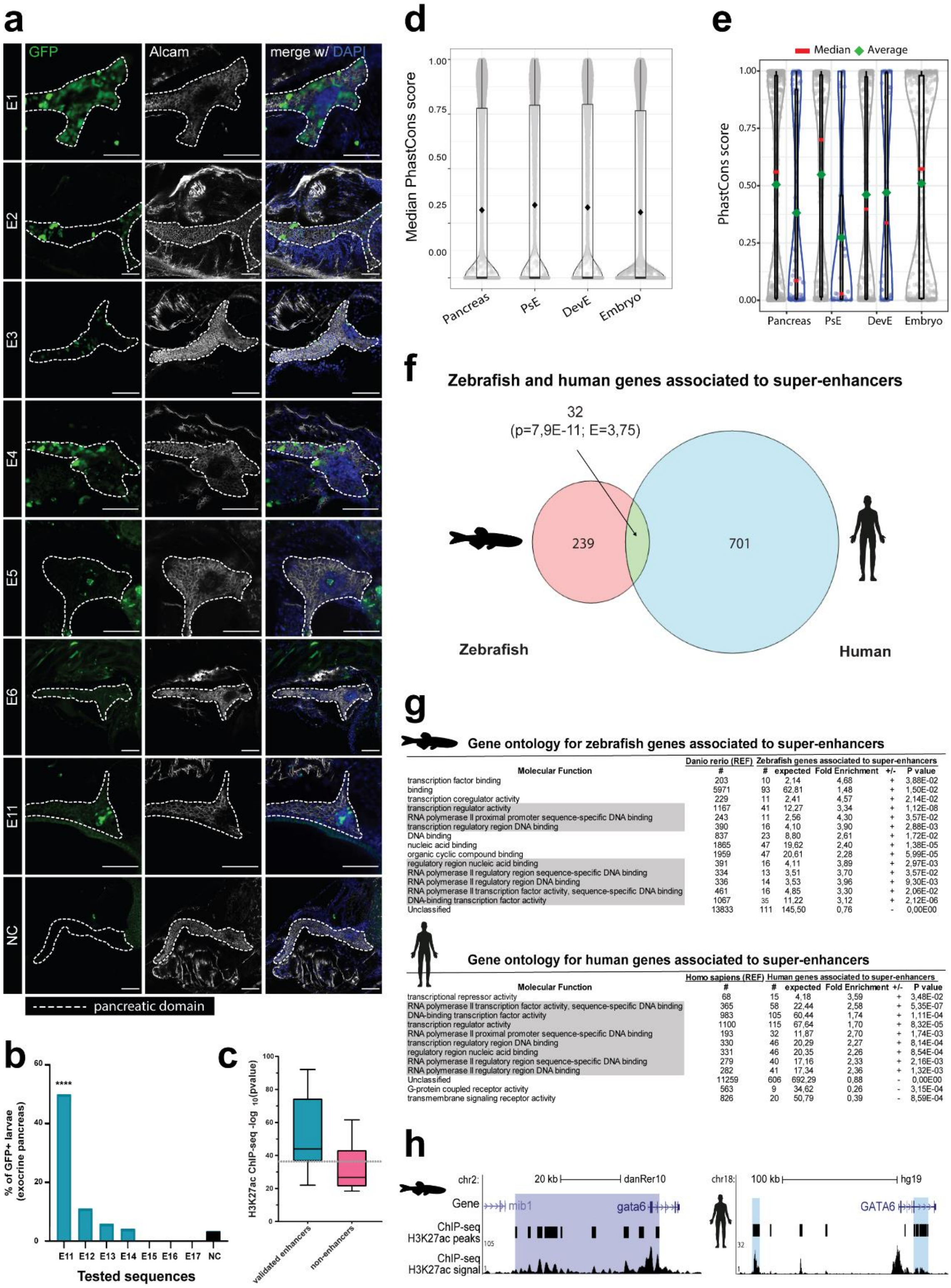

Gene ontology for zebrafish genes associated to super-enhancers

Molecular Function	Danio rerio (REF) #	Zebrafish genes associated to super-enhancers #	expected	Fold Enrichment	+/-	P value
transcription factor binding	203	10	2,14	4,68	+	3,88E-02
binding	5971	93	62,81	1,48	+	1,50E-02
transcription coregulator activity	229	11	2,41	4,57	+	2,14E-02
transcription regulator activity	1167	41	12,27	3,34	+	1,12E-08
RNA polymerase II proximal promoter sequence-specific DNA binding	243	11	2,56	4,30	+	3,57E-02
transcription regulatory region DNA binding	390	16	4,10	3,90	+	2,88E-03
DNA binding	837	23	8,80	2,61	+	1,72E-02
nucleic acid binding	1865	47	19,62	2,40	+	1,38E-05
organic cyclic compound binding	1959	47	20,61	2,28	+	5,99E-05
regulatory region nucleic acid binding	391	16	4,11	3,89	+	2,97E-03
RNA polymerase II regulatory region sequence-specific DNA binding	334	13	3,51	3,70	+	3,57E-02
RNA polymerase II regulatory region DNA binding	336	14	3,53	3,96	+	9,30E-03
RNA polymerase II transcription factor activity, sequence-specific DNA binding	461	16	4,85	3,30	+	2,06E-02
DNA-binding transcription factor activity	1067	35	11,22	3,12	+	2,12E-06
Unclassified	13833	111	145,50	0,76	-	0,00E00

Gene ontology for human genes associated to super-enhancers

Molecular Function	Homo sapiens (REF) #	Human genes associated to super-enhancers #	expected	Fold Enrichment	+/-	P value
transcriptional repressor activity	68	15	4,18	3,59	+	3,48E-02
RNA polymerase II transcription factor activity, sequence-specific DNA binding	365	58	22,44	2,58	+	5,35E-07
DNA-binding transcription factor activity	983	105	60,44	1,74	+	1,11E-04
transcription regulator activity	1100	115	67,64	1,70	+	8,32E-05
RNA polymerase II proximal promoter sequence-specific DNA binding	193	32	11,87	2,70	+	1,74E-03
transcription regulatory region DNA binding	330	46	20,29	2,27	+	8,14E-04
regulatory region nucleic acid binding	331	46	20,35	2,26	+	8,54E-04
RNA polymerase II regulatory region sequence-specific DNA binding	279	40	17,16	2,33	+	2,16E-03
RNA polymerase II regulatory region DNA binding	282	41	17,34	2,36	+	1,32E-03
Unclassified	11259	606	692,29	0,88	-	0,00E00
G-protein coupled receptor activity	563	9	34,62	0,26	-	3,15E-04
transmembrane signaling receptor activity	826	20	50,79	0,39	-	8,59E-04

Supplementary Fig. 3. In vivo enhancer validation and comparisons between Human and Zebrafish putative pancreatic enhancers. a-c *In vivo* validation of selected putative pancreatic enhancers by transgenic zebrafish reporter assays, identified by ATAC and H3k27ac ChIP-seq data. **a** Representative confocal images of F0 transgenic zebrafish larvae for all validated enhancers (E1-E6 and E11, n values are discriminated in Supplementary Dataset 4); whole mount immunohistochemistry of 10-12 dpf zebrafish larvae showing GFP reporter expression (green), within the pancreatic domain (dashed white line). The empty vector was used as negative control (NC). The exocrine pancreas is stained with anti-Alcam (white) antibody and nuclei are stained with DAPI. Scale bar: 60 μm. **b** Percentage of F0 transgenic zebrafish larvae with GFP expression within the exocrine pancreas for sequences with low H3K27ac ChIP signal value: (($-\log_{10}$(*p*-value)<35) (sequences E11 to 17). Two-sided chi-square test with Yates' correction, *p*-value<0.05 were considered significant (*****p*<0.0001). The empty vector was used as negative control (NC). The exact *p*-value are discriminated in Supplementary Dataset 4. **c** H3K27ac ChIP-seq signal [H3K27ac ChIP-seq -log10(p-value)] of validated pancreatic enhancer sequences (validated enhancers) versus tested sequences without enhancer activity in the differentiated pancreas (non-enhancers; centre, median; box, upper and lower quartile; whiskers, minimum and maximum value). Prior to enhancer validation we divided the sequences into two groups based on their H3K27ac ChIP-seq signal; “high H3K27ac” [sequences corresponding to the top 10 higher values of H3K27ac ChIP-seq -log10(p-value)] and “low H3K27ac” (the remaining 7 sequences). The dashed grey line represents the threshold between the “high” [-log10(p-value) from 36.5 to 92.1] and “low H3K27ac" groups [-log10(p-value) from 18.5 to 28.4] **d** Distribution of the median PhastCons scores for each zebrafish putative enhancer sequence active in adult pancreas (14301), adult pancreas only (PsE, 6918), adult pancreas and embryo (DevE, 8368) and

in embryo (Embryo, 65871). Putative active enhancer sequences converted from DanRer10 to DanRer7 (liftOver) to match the available conservation scores for zebrafish and 7 vertebrates. The median value is zero for the 4 enhancer sets (lower bar of the boxplots) since most sequences are not conserved, while the average is, respectively, 0.31, 0.33, 0.32, 0.30 (back diamond inside the boxplot). **e** PhastCons scores (99 vertebrate genomes against hg38) for human sequences converted from zebrafish putative enhancers filtered by ATAC-seq peaks. Grey dots: conserved sequences not overlapping the H3K27ac mark in human pancreas (512 pancreas, 258 PsE, 254 DevE and 334 embryo). Blue dots: conserved sequences also showing H3K27ac signal in human pancreas (ENCODE data; 73 pancreas, 33 PsE, 40 DevE). Green diamonds: average (grey dots: 0.50, 0.55, 0.46, 0.51; blue dots: 0.38, 0.27, 0.47, respectively for pancreas, PsE, DevE and embryo). Red horizontal line: median (grey dots: 0.6, 0.7, 0.4, 0.6; blue dots: 0.09, 0.03, 0.34, respectively for pancreas, PsE, DevE and embryo). **f** Zebrafish and human genes associated with super-enhancers, identified by ROSE[56]. Statistical significance was calculated by hypergeometric enrichment test, *p*-value (p) and the enrichment are represented. **g** Gene ontology for genes associated with super-enhancers in zebrafish (above) and human (below). **h** H3K27ac profile of the landscape of a gene important in pancreatic development in zebrafish (*gata6*; left) and human (*GATA6*; right); super-enhancers are highlighted in purple (zebrafish) or blue (human). Data included in Source Data file for **b-g**

Supplementary figure 4

a

PsE			C1			C2			C3			C4		
Motif	Name	P-value	Motif	Name	P-value	Motif	Name	P-value	Motif	Name	P-value	Motif	Name	P-value
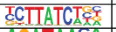	GATA6	1E-96	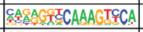	HNF4a	1E-60	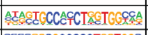	CTCF	1E-24	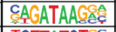	GATA1	1E-32	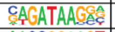	GATA1	1E-29
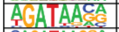	GATA3	1E-94	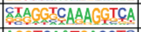	PPARa	1E-51	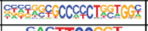	BORIS	1E-19	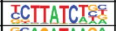	GATA6	1E-31	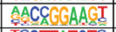	ETV1	1E-21
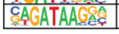	GATA1	1E-91	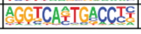	FXR	1E-48	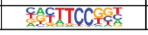	FLI	1E-13	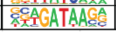	GATA4	1E-31	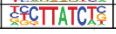	GATA2	1E-21

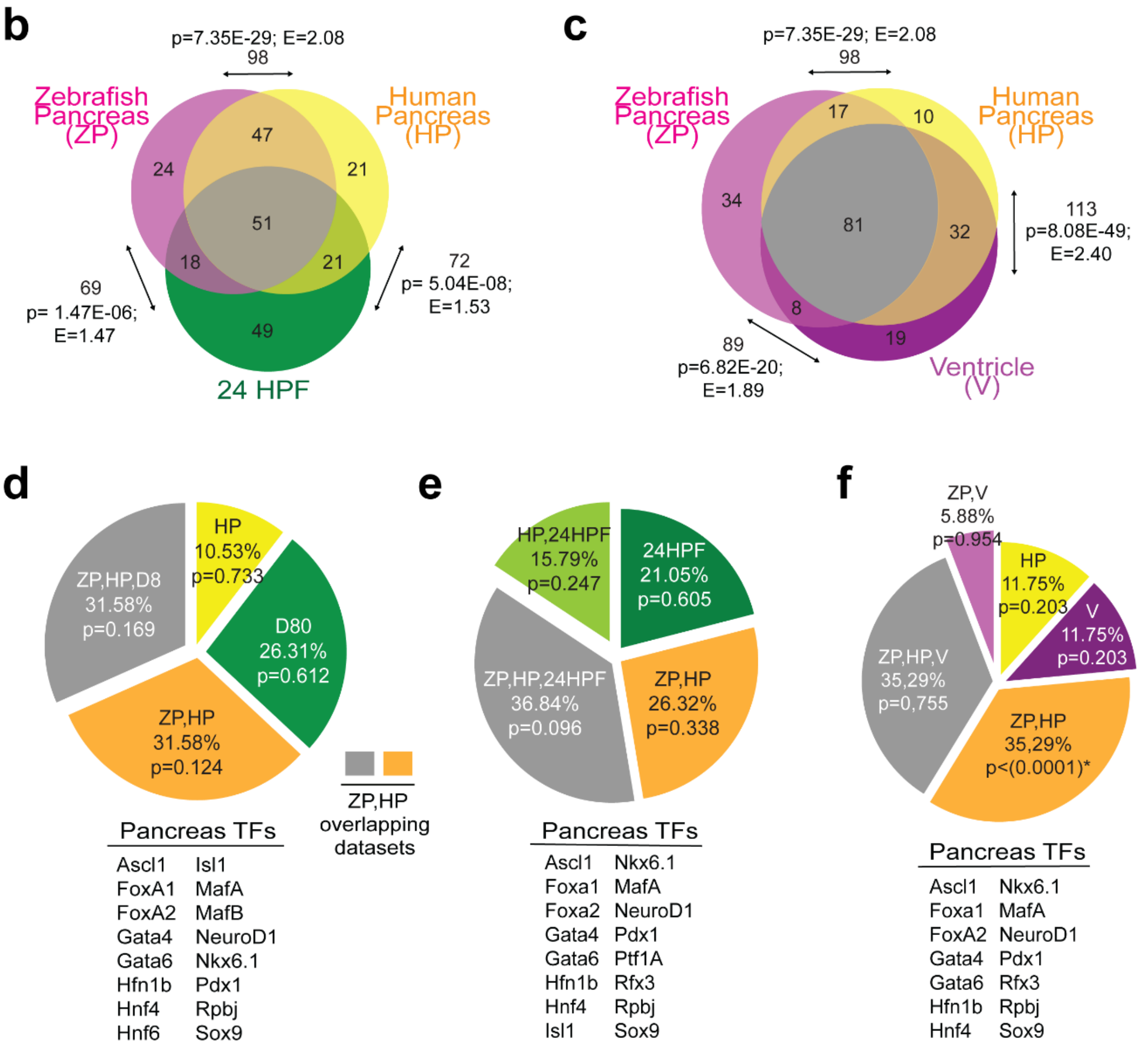

Supplementary Fig. 4. Zebrafish and human pancreatic enhancers share TFBS. a List of top three TFBS motifs enriched in H3K27ac ChIP-seq data, for the different enhancer sets: pancreas specific enhancers (PsE) and clusters of developmental enhancers C1, C2, C3 and C4, with the respective *p*-value calculated by HOMER (Two-sided hypergeometric enrichment test)[100]. **b-c** Regions enriched in ATAC-seq and H3K27ac signal from zebrafish pancreas (ZP), human pancreas (HP), 24hpf zebrafish embryos (24HPF) and human ventricle (V) were investigated for TFBS motifs (Supplementary Dataset 3t-u). The top 140 enriched TFBS motifs from each tissue were selected and the overlap of those sets was analyzed. Arrows: number of TFBS motifs shared between two different groups, the enrichment of TFBS motifs and respective *p*-value for each intersection. Statistical significance was determined by hypergeometric enrichment test. Number of TFBS motifs identified in each group/intersection. The exact *p*-value and enrichment are described in the figure. **b** ZP, HP and 24HPF; **c** ZP, HP, V. **d-f** The motifs corresponding to 25 pancreas transcription factors (Pancreas TFs) selected from literature were analyzed for their representation in H3K27ac peaks filtered with ATAC-seq peaks among the different zebrafish (ZP, 24HPF, D80) and human tissues (HP, V), and tissue-intersections shown in b-c). The distribution of these TFBS motifs through the different tissues is shown along with the percentage and the respective *p-value* indicated for each group: **d** ZP, HP and dome+80%epiboly (D80); **e)** ZP, HP and 24hpf; **f)** ZP, HP and V. The list of TFs presents in the tissues, for each graph, is depicted below. Statistical significance was assessed by two-sided Fisher exact test, *p-values*<0.007 were considered significant (Bonferroni correction). The exact *p*-values are discriminated in the graph. Data included in Source Data file for **a-f**.

Supplementary figure 5

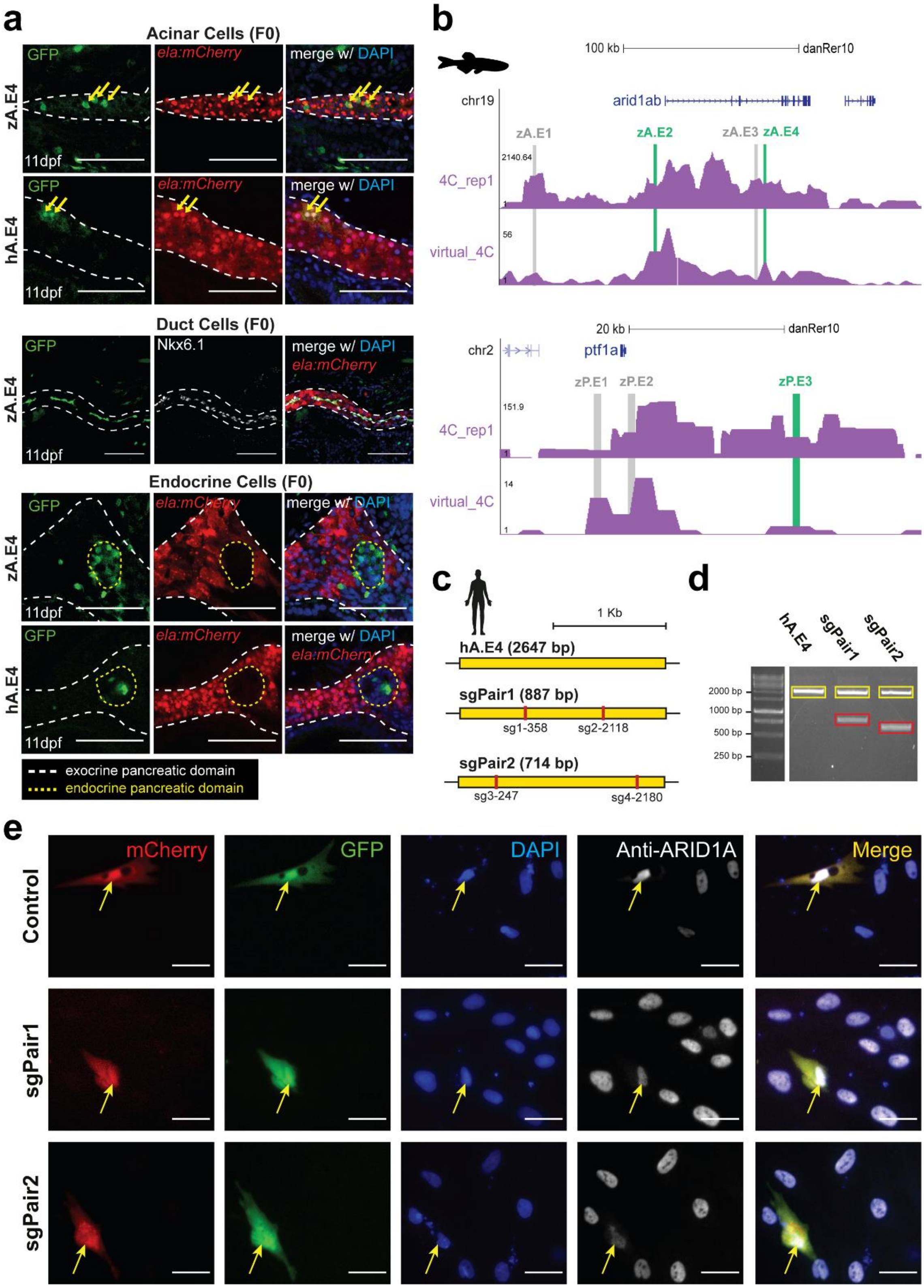

Supplementary Fig. 5. Human and zebrafish ARID1A/arid1ab enhancer reporter assays and CRISPR-Cas9-mediated deletion of the zA.E4 in a human ductal cell line. **a** Human and zebrafish *ARID1A/arid1ab* enhancers drive expression in various pancreatic cell types. Representative confocal images of 11dpf Tg(*ela:mCherry*) zebrafish larvae injected with Z48 vector carrying the zebrafish zA.E4 or human hA.E4 enhancer. The top panel shows elastase-producing acinar cells (red; zA.E4 n=28 ; hA.E4 n=39), the middle panel shows Nkx6.1-positive duct cells (white; zA.E4 n=10 ; hA.E4 n=22), and the bottom panel shows the endocrine pancreas domain (yellow dashed line; zA.E4 n=26 ; hA.E4 n=39). In all panels, the exocrine pancreas domain is indicated with a white dashed line and nuclei are stained with DAPI (blue). Scale bar: 60 µm. **b** Genomic landscape of zebrafish *arid1ab* gene (top) and *ptf1a* gene (bottom), showing *arid1ab* 4C interactions (purple) from 4C-seq replicates and virtual 4C from HiChIP for H3K4me3 in adult zebrafish pancreas. Tested putative enhancers are highlighted by the colored boxes (grey and green). **c** Schematic depiction of the targeting strategy for deletion of the hA.E4 locus. The CRISPR sgRNA target sites are depicted in red. **d** Agarose gel showing the wild-type (yellow) and deleted (red) PCR amplified hA.E4 sequence after gene editing with each respective sgRNA pair (n=3). **e** Representative fluorescent microscopy images of transfected hTERT-HPNE human cells (n=3), defined by the co-expression of GFP (green) and mCherry (green). Nuclei are stained with DAPI (blue) and anti-ARID1A antibody (white). The yellow arrows indicate the double transfected cell. Images were captured with Leica DMI6000 FFW microscope. Scale bar: 40 µm. Abbreviations: ela, elastase.

Supplementary figure 6

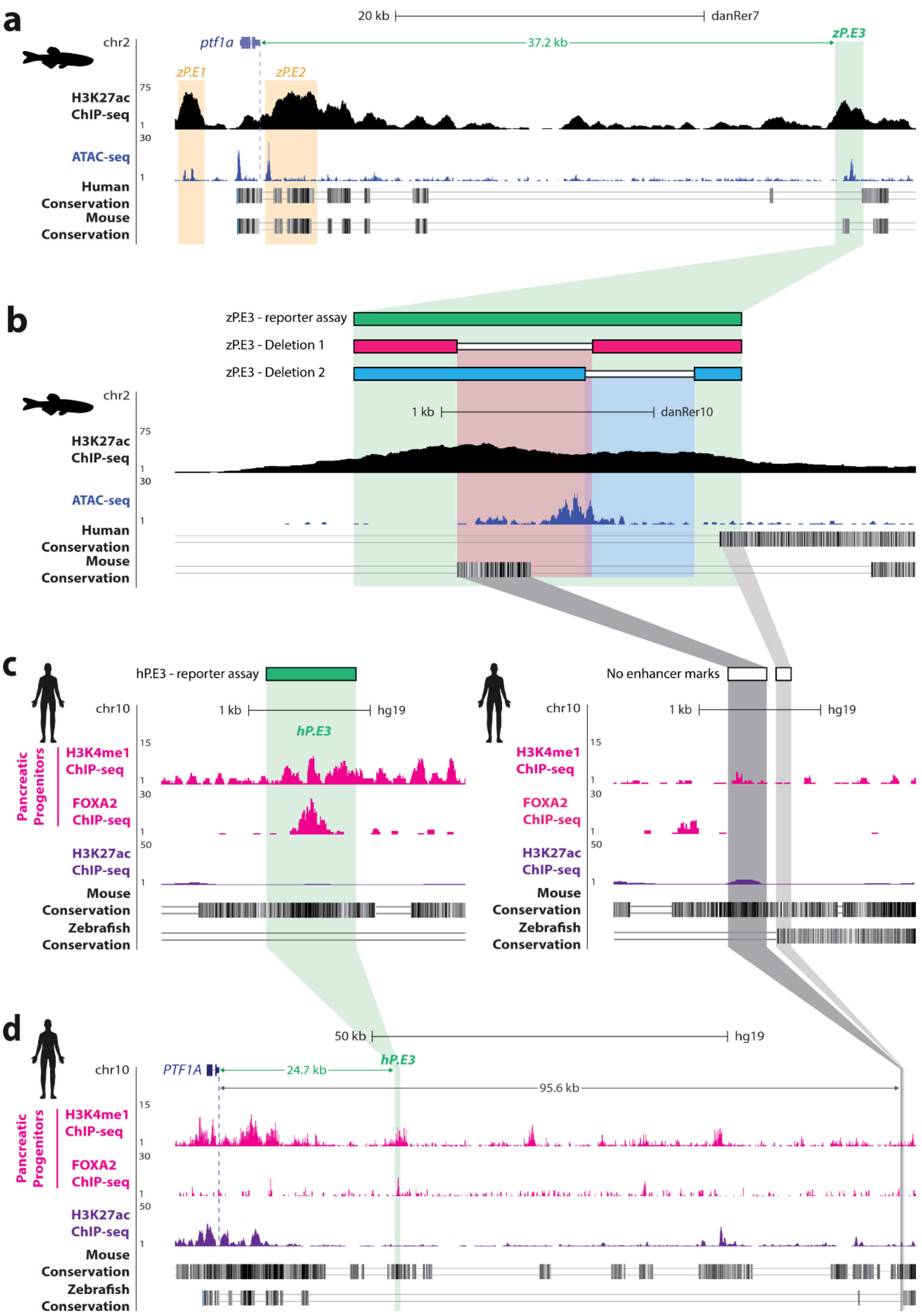

Supplementary Fig.6. Regions of sequence conservation within the zP.E3 enhancer sequence. a UCSC Genome Browser view of zP.E3 showing H3K27ac ChIP-seq (black) and ATAC-seq (blue) from whole zebrafish pancreas (above), and the conservation tracks (below) displaying where the human and mouse genomes aligns to the zebrafish sequence (darker shading indicates higher BLASTZ scores; white indicates no alignment). The validated distal *ptf1a* enhancer (zP.E3) is indicated by the green box and other validated enhancers by the orange boxes. **b** Zoom-in of the zP.E3 region depicted in a) and schematic depiction of the generated zP.E3 deletion alleles; Deletion1 and Deletion2. The zP.E3 sequence contains a 332bp region conserved with mouse (dark grey) and a 120bp region conserved with human (light grey). **c** UCSC Genome Browser view of the validated hP.E3 (left panel) and the regions depicted in b) [the region conserved between zebrafish and mouse (dark grey) and the region conserved between zebrafish and human (light gray)] (right panel), showing H3K4me1 and FOXA2 ChIP-seq from pancreatic progenitor cells (pink), H3K27ac ChIP-seq from adult pancreatic tissue (purple) and conservation tracks for mouse and zebrafish (below). **d** Zoom-out of the regions depicted in c), showing the full *PTF1A* landscape.

Supplementary figure 7

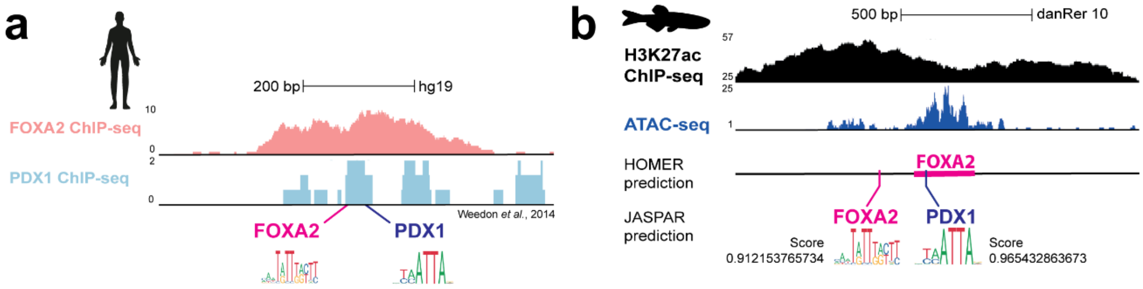

Supplementary Fig. 7. *In vivo* enhancer validation and comparisons between Human and Zebrafish putative pancreatic enhancers. a) ChIP-seq density plots at the hP.E3 locus showing FOXA2 (pink) and PDX1 (blue) ChIP-seq peaks generated from human endocrine islets[2]. The location of the respective predicted binding sites is depicted below. **b)** H3K27ac ChIP-seq (black) and ATAC-seq (blue) read density plots at the zP.E3 locus, and putative FOXA2 and PDX1 transcription factor binding sites predicted by JASPAR and HOMER[100] with respective score.

Supplementary figure 8

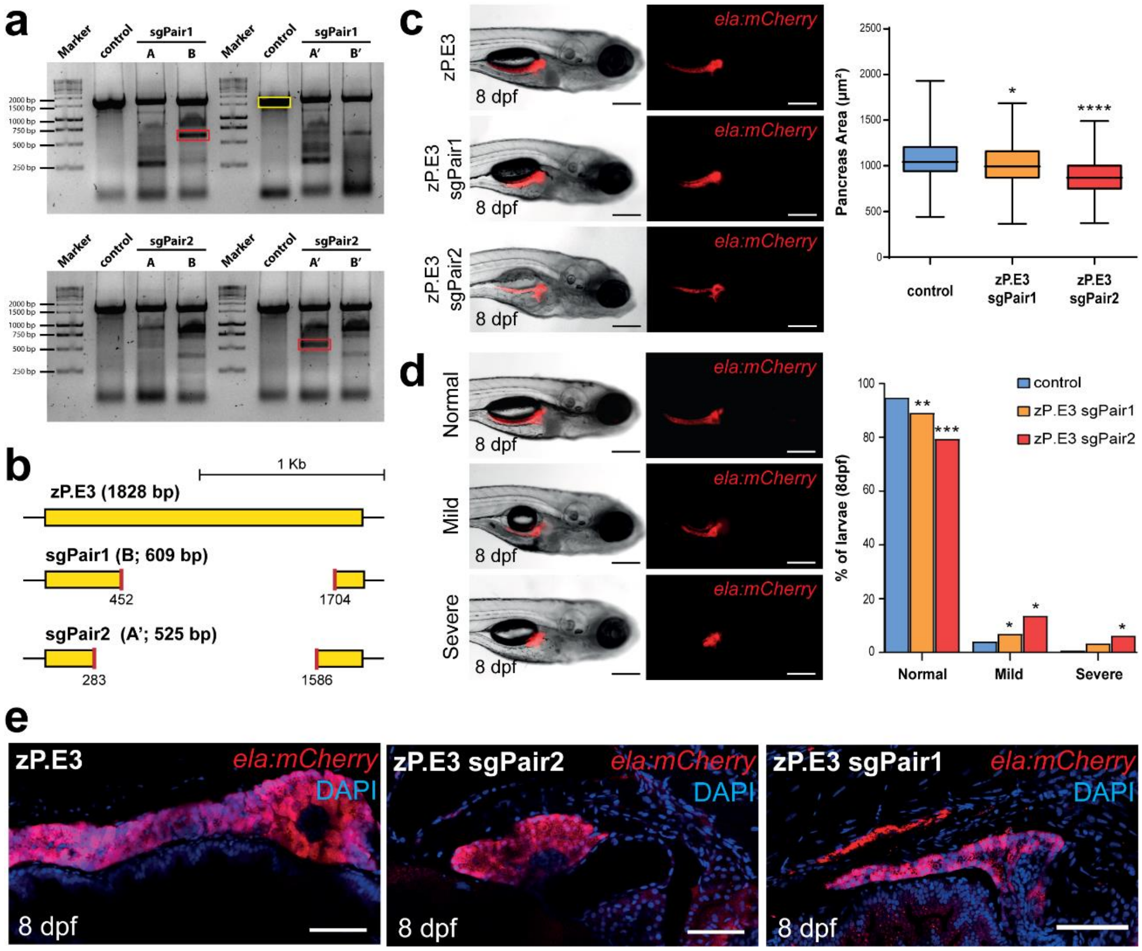

Supplementary Fig. 8. CRISPR-Cas9-mediated deletion of the zP.E3 enhancer impairs pancreas development. a PCR screening of zP.E3 deletion after co-injection of zebrafish embryos with Cas9 and different combinations of sgRNAs: successful deletions appear as truncated PCR products (red box), in comparison with the wild-type sequence from non-injected embryos (control, yellow box). A, B; A', B' represent different batches of embryos injected with each pair of sgRNAs (n=5 independent injections per sgRNA pair). **b** Schematic representation of the deletions induced by CRISPR-Cas9 depicted in a) (yellow and red boxes). **c** Tg(*ela:mCherry*) embryos were injected with Cas9 alone (zP.E3) or co-injected with Cas9 and a pair of sgRNAs (zP.E3 sgPair1 or zP.E3 sgPair2) and monitored at 8dpf (Cas9 alone, n=140; zP.E3 sgPair1, n=110; zP.E3 sgPair2, n=108 larvae, pooled from 3 independent experiments each). Representative live images are shown in the left panels. Scale bar: 250 µm. The quantification of total pancreas area is represented in the right panel (centre, median; box, upper and lower quartile; whiskers, minimum and maximum value). Unpaired student's t-test (two-tailed), *p-values*<0.05 were considered significant ((**p*= 0.0107, *****p*=1.1486×10E-11). **d** The injected F0 8dpf larvae from c) were classified as either normal or as one of the two following classes: mild pancreatic defect characterized by significantly reduced pancreas size (mild), or severe pancreatic defects characterized by a reduced pancreas with absence of the pancreatic tail (severe). Representative live images of each pancreatic phenotype are shown in the left panels. Scale bar: 250 µm. The percentage of larvae in each phenotypic class is represented in the right panel and the n described in **c**. Fisher's exact test (two-sided), *p*-values<0.05 were considered significant (*p*-values, left to right: ***p*=0.0083, ****p*=0.0002, **p*=0.0184, *p*=0.0102, and *p*=0.0229). **e** Representative confocal fluorescent images of 8dpf Tg(*ela:mCherry*) larvae showing impaired development of pancreas upon injection of Cas9 and the respective sgPairs targeting the zP.E3 enhancer

(zP.E3 sgPair1 or zP.E3 sgPair2), in comparison to the control, injected with Cas9 alone (zP.E3). This experiment, independent of the 3 replicates presented in c-d), produced the following results: zP.E3 (100% normal pancreas in a total of n=18 larvae), zP.E3 sgPair1 (8.33% of pancreatic phenotypes in a total of n=12 larvae) and zP.E3 sgPair2 (18.75% of pancreatic phenotypes in a total of n=16 larvae). Nuclei are stained with DAPI (blue) and acinar cells are labeled with mCherry (red). Scale bar: 60 µm. Abbreviations: ela, elastase. Data included in Source Data file for **c**.

Supplementary figure 9

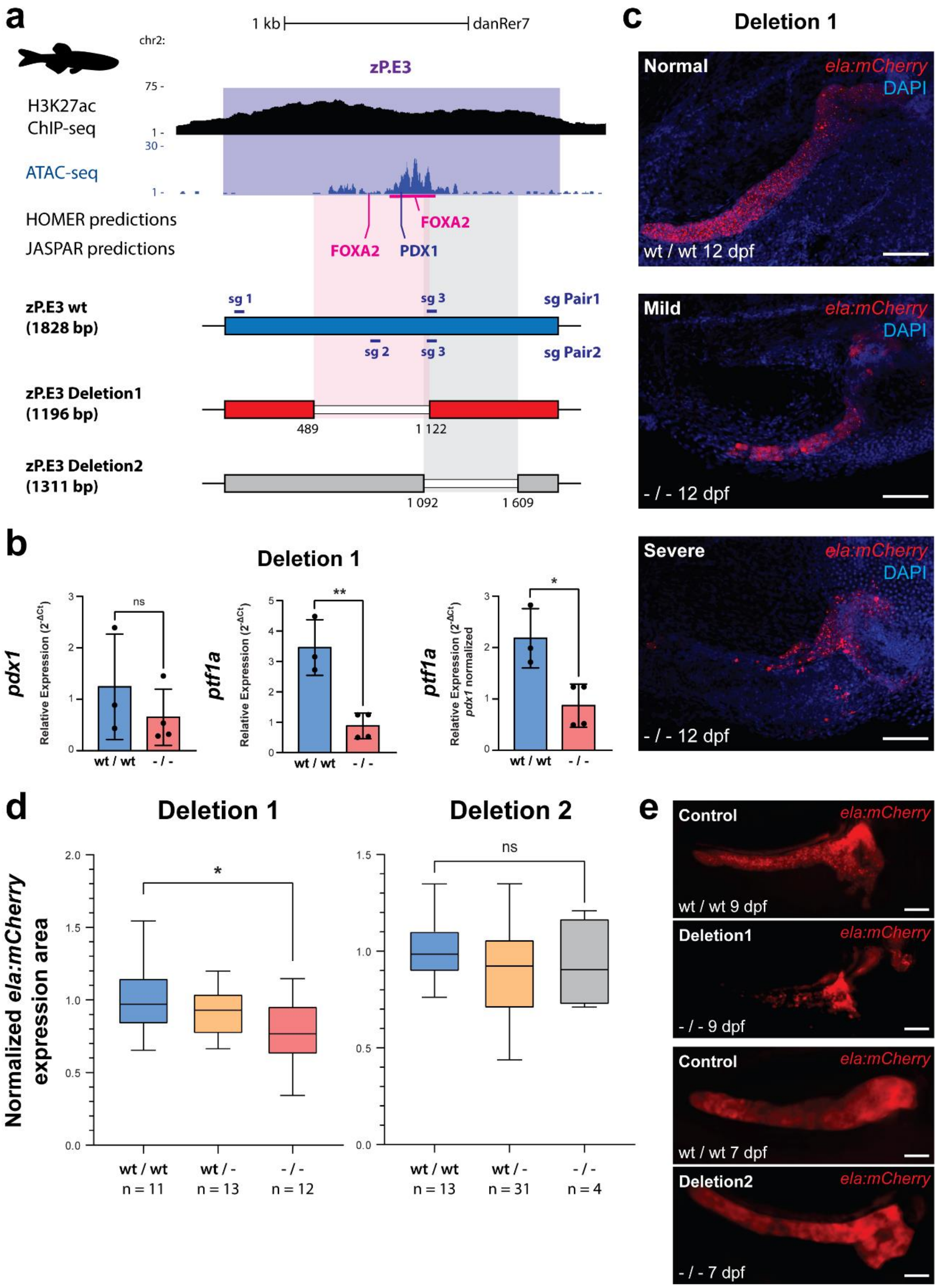

Supplementary Fig.9. Independent deletions of the zP.E3 enhancer have distinct phenotypic outcomes. a UCSC Genome Browser view of zP.E3 showing H3K27ac ChIP-seq (black) and ATAC-seq (blue) from whole zebrafish pancreas samples (upper panels), along with the location of predicted TFBS for FOXA2 and PDX1 (middle panel), schematic depiction of the sgRNA pairs and the generated zP.E3 deletion alleles: Deletion1 (generated with sgPair1) and Deletion2 (generated with sgPair2). **b** Relative expression of *pdx1* (left panel) and *ptf1a* (middle panel) in pancreatic progenitor cells of 48hpf embryos, and corresponding *pdx1*-normalized *ptf1a* expression (right panel). Each biological replicate was obtained from a batch of 30 embryos. Unpaired student's t-test (two-tailed), *p-values*<0.05 were considered significant (***p*=0.0039, **p*=0.0172). **c** Representative confocal images (maximum intensity projections) of 12dpf Tg(*ela:mCherry*) larvae showing impaired development of pancreas in Deletion1 homozygous larva (-/-) compared to the control (wt/wt sibling). Larvae resulted from a single incross of heterozygous animals and only the homozygous larvae (wt/wt and -/-) were selected for confocal imaging: wt/wt, n=3 (100% normal phenotypes); -/-, n=7 (57.14% normal, 14.29% mild, and 28.57% severe phenotypes). Nuclei are stained with DAPI (blue) and acinar cells with mCherry (red). Scale bar: 60 µm. **d** Normalized area of *ela:mCherry* expression of Tg(*ela:mCherry*) Deletion1 and Deletion2 homozygous (-/-) and heterozygous (wt/-) larva (9 and 7dpf, respectively), compared to the respective control (wt/wt siblings; centre, median; box, upper and lower quartile; whiskers, minimum and maximum value). Individual values were normalized to the mean of their respective control group. Unpaired student's t-test (two-tailed), *p-values*<0.05 were considered significant (**p*=0.0353). **e** Representative live image of the pancreas of Tg(*ela:mCherry*) Deletion1 and Deletion2 homozygous larva (-/-) (9 and 7dpf, respectively), compared to a control larva (wt/wt sibling). From top to bottom: 9dpf

wt/wt, n= 11 (100% normal phenotypes); 9dpf Deletion1 -/-, n= 12 (33.33 normal, 33.33 mild, and 33.33 severe phenotypes); 7dpf wt/wt, n=13 (100% normal phenotypes); 7dpf Deletion1 -/-, n=4 (100% normal phenotypes). Scale bar: 60 µm. Abbreviations: ela, elastase; ins, insulin. Data included in Source Data file for **b** and **d**.

Supplementary figure 10

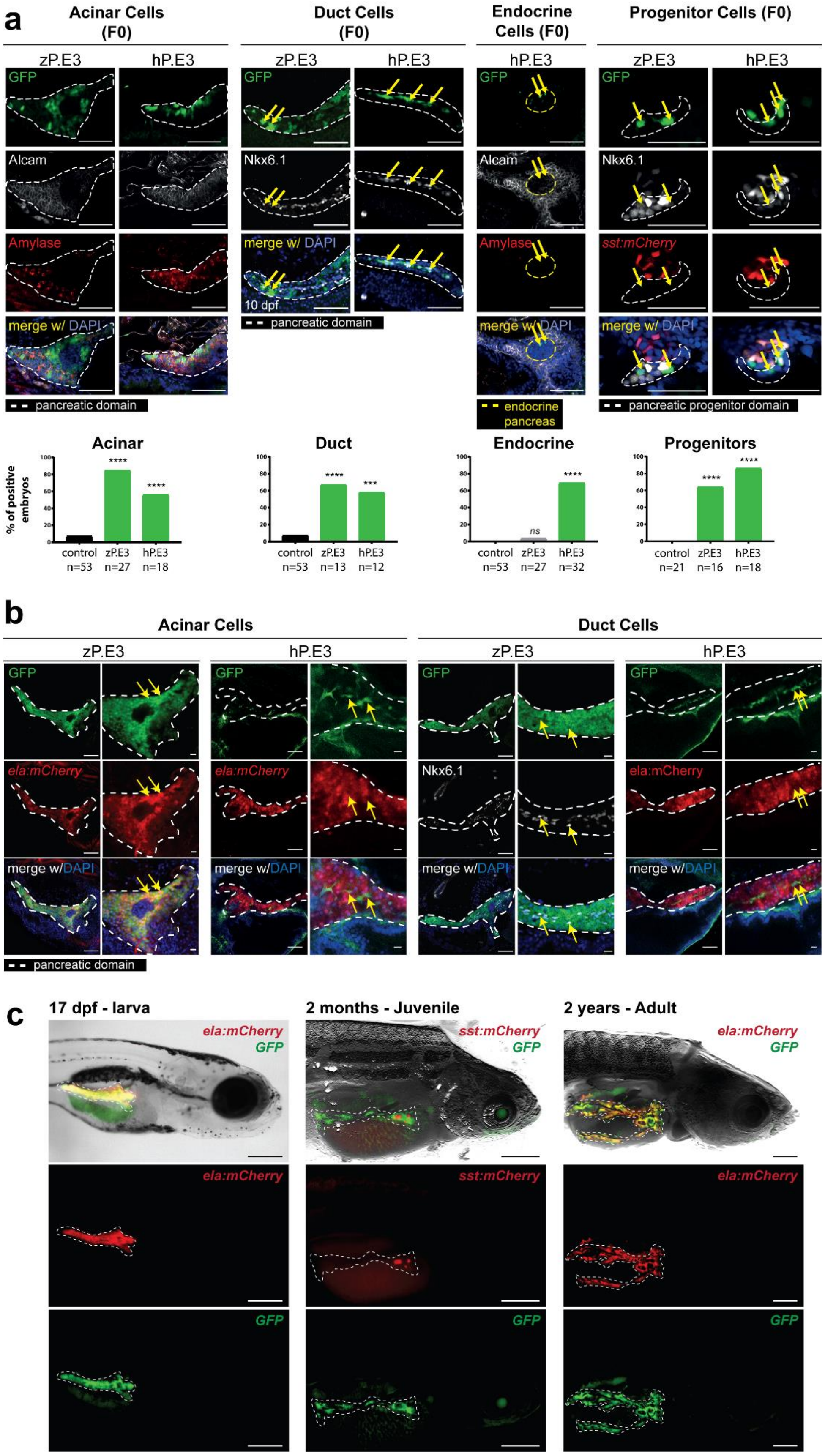

Supplementary Fig. 10. Human and zebrafish distal *PTF1a/ptf1a* enhancers drive similar reporter expression in various pancreatic cell types. a Left panels: Representative confocal images of GFP expression (green) driven by zebrafish zP.E3 or human hP.E3 sequences in F0 10dpf zebrafish larvae in pancreatic acinar cells, stained with anti-Alcam (white) and anti-Amylase (red), duct cells, stained with anti-Nkx6.1 (white) and cells of the principal islet of the endocrine pancreas, which lack staining of acinar cell markers. Right panel: Representative confocal images of 48hpf F0 zebrafish embryos showing zP.E3 and hP.E3-driven GFP expression within the pancreatic progenitor domain. Pancreatic progenitor cells are identified by anti-Nkx6.1 staining (white), adjacent to the principal islet marked by somatostatin expression in differentiated delta-cells (red). Nuclei are stained with DAPI (blue). Yellow arrows point to examples of GFP expression in each pancreatic cell type. Scale bar: 60 μm. The corresponding percentage of zebrafish embryos showing zP.E3 or hP.E3-mediated GFP expression in transient transgenesis assays are depicted below. Statistical significance determined by Fisher's exact test (two-sided), p-values<0.05 were considered significant (p-values, left to right: ****p=3.387×10-12; ****p=5.643×10-5; ****p=8.00×10-5; ***p=0.0005; ****p=5.10×10-13; ****p=2.299×10-5; ****p=2.586×10-5). **b** Left Panels: Representative confocal images of Tg(*zP.E3:GFP*, *ela:mCherry*) and Tg(*hP.E3:GFP*, *ela:mCherry*) F1 larvae, showing co-localization of zP.E3 and hP.E3 mediated GFP expression (green) and acinar cell-specific mCherry expression (red). Right Panels: Representative confocal images of Tg(*zP.E3:GFP*) and Tg(*hP.E3:GFP, ela:mCherry*) F1 larvae showing GFP expression in the duct cells of the exocrine pancreas. In Tg(*zP.E3:GFP*) larvae duct cells are stained with anti-Nkx6.1 (white), while in Tg(*hP.E3:GFP, ela:mCherry*) larvae duct cells appear as mCherry-negative cells within the exocrine pancreatic tissue. Tg(*zP.E3:GFP, ela:mCherry*) larvae: 100% of larvae

show co-localization of GFP with acinar specific mCherry expression (n=8 pooled from two impendent experiments) or with duct specific Nkx6.1 staining (n=8). Tg(*hP.E3:GFP*, *ela:mCherry*) larvae: of 2 analyzed larvae (n=2) 100% show GFP expression in duct cells and acinar cells, although, in the case of acinar cells, in a very reduced number of cells. Nuclei are labeled with DAPI (blue). Yellow arrows point to examples of GFP-positive duct cells. Scale bar 60 μm. **c** Zebrafish zP.E3 reporter transgenic line drives GFP expression in the exocrine pancreas, from larva to adult. Representative images of zP.E3-driven GFP expression (green) in 17dpf Tg(*ela:mCherry*) larvae (n=1; scale bar: 100 μm), 2 months Tg(*sst:mCherry*) juvenile (n=1), and 2 year old Tg(*ela:mCherry*) adult (scale bar: 500 μm), showing sustained enhancer activity in the exocrine pancreatic tissue (n=1;delimited by the white dashed line). mCherry is represented in red. Abbreviations: ela, elastase; sst, somatostatin. Data included in Source Data file for **a**.
