# Supplementary material for: Multidimensional chromatin profiling of zebrafish pancreas to uncover and investigate disease-relevant enhancers
Source: Nat Commun. 2022 Apr 11;13:1945. doi: 10.1038/s41467-022-29551-7 (PMC9001708; doi:10.1038/s41467-022-29551-7)
Supplement: Supplementary file 3 — Supplementary data1-17 [file 41467_2022_29551_MOESM3_ESM.zip › SupplementaryFile1_FASTQC_reports/Supplementary data 1_Pancreas H3K27ac ChIP-seq fastqc 1.html]

chip1.fastq FastQC Report 

FastQC Report

quarta 20 nov 2019  
chip1.fastq

## Summary

- Basic Statistics
- Per base sequence quality
- Per tile sequence quality
- Per sequence quality scores
- Per base sequence content
- Per sequence GC content
- Per base N content
- Sequence Length Distribution
- Sequence Duplication Levels
- Overrepresented sequences
- Adapter Content
- Kmer Content

## Basic Statistics

| Measure | Value |
| --- | --- |
| Filename | chip1.fastq |
| File type | Conventional base calls |
| Encoding | Sanger / Illumina 1.9 |
| Total Sequences | 19453108 |
| Sequences flagged as poor quality | 0 |
| Sequence length | 49 |
| %GC | 39 |

## Per base sequence quality

## Per tile sequence quality

## Per sequence quality scores

## Per base sequence content

## Per sequence GC content

## Per base N content

## Sequence Length Distribution

## Sequence Duplication Levels

## Overrepresented sequences

No overrepresented sequences

## Adapter Content

## Kmer Content

| Sequence | Count | PValue | Obs/Exp Max | Max Obs/Exp Position |
| --- | --- | --- | --- | --- |
| TCGGAAG | 4135 | 0.0 | 5.7688675 | 43 |
| GATCGGA | 5225 | 0.0 | 5.4291363 | 43 |
| ATCGGAA | 4820 | 0.0 | 5.35029 | 43 |
| CGGAAGA | 4215 | 0.0 | 5.149522 | 43 |

Produced by FastQC (version 0.11.5)
