# Supplementary material for: Multidimensional chromatin profiling of zebrafish pancreas to uncover and investigate disease-relevant enhancers
Source: Nat Commun. 2022 Apr 11;13:1945. doi: 10.1038/s41467-022-29551-7 (PMC9001708; doi:10.1038/s41467-022-29551-7)
Supplement: Supplementary file 3 — Supplementary data1-17 [file 41467_2022_29551_MOESM3_ESM.zip › SupplementaryFile1_FASTQC_reports/Supplementary data 10_RNA-seq Endocrine old fastqc 1 .html]

FCHGVKNBBXX-HKZEBggcRAADRAAPEI-206\_L3\_1.fq FastQC Report 

FastQC Report

Wed 5 Jul 2017  
FCHGVKNBBXX-HKZEBggcRAADRAAPEI-206\_L3\_1.fq

## Summary

- Basic Statistics
- Per base sequence quality
- Per tile sequence quality
- Per sequence quality scores
- Per base sequence content
- Per sequence GC content
- Per base N content
- Sequence Length Distribution
- Sequence Duplication Levels
- Overrepresented sequences
- Adapter Content
- Kmer Content

## Basic Statistics

| Measure | Value |
| --- | --- |
| Filename | FCHGVKNBBXX-HKZEBggcRAADRAAPEI-206\_L3\_1.fq |
| File type | Conventional base calls |
| Encoding | Sanger / Illumina 1.9 |
| Total Sequences | 39591655 |
| Sequences flagged as poor quality | 0 |
| Sequence length | 50 |
| %GC | 49 |

## Per base sequence quality

## Per tile sequence quality

## Per sequence quality scores

## Per base sequence content

## Per sequence GC content

## Per base N content

## Sequence Length Distribution

## Sequence Duplication Levels

## Overrepresented sequences

| Sequence | Count | Percentage | Possible Source |
| --- | --- | --- | --- |
| CTTTGGTGTTCCTGGTGCTCCTTGGAGCTGCCTTTGCTCTGGATGATGAC | 57829 | 0.1460636086064096 | No Hit |
| CACCATTCCTCGCCTCTGCTTCGAGAACAGTGTGACCATGGCAGTGTGGC | 53250 | 0.13449804005414778 | No Hit |

## Adapter Content

## Kmer Content

| Sequence | Count | PValue | Obs/Exp Max | Max Obs/Exp Position |
| --- | --- | --- | --- | --- |
| GAATCGG | 7830 | 0.0 | 21.29546 | 4 |
| AGAATCG | 8570 | 0.0 | 19.687487 | 3 |
| AATCGGT | 8420 | 0.0 | 19.620382 | 5 |
| ACGCGTA | 3230 | 0.0 | 16.54938 | 22 |
| ATCGGTT | 10780 | 0.0 | 15.835163 | 6 |
| GTTCAAT | 10210 | 0.0 | 15.128259 | 1 |
| TTCAATC | 12300 | 0.0 | 14.385875 | 2 |
| CGGTTTT | 11565 | 0.0 | 14.341855 | 8 |
| CTTACCC | 7600 | 0.0 | 14.313637 | 1 |
| GAGTTAA | 7165 | 0.0 | 13.815674 | 3 |
| TGCCGTA | 11055 | 0.0 | 13.730605 | 31 |
| TCGGTTT | 12835 | 0.0 | 13.608311 | 7 |
| CACGCGT | 4040 | 0.0 | 13.23131 | 21 |
| CGGCGAT | 8385 | 0.0 | 13.21044 | 1 |
| CAATCGG | 2055 | 0.0 | 13.166576 | 4 |
| ACGATCC | 3830 | 0.0 | 13.152625 | 3 |
| CAAGAAT | 15475 | 0.0 | 12.890044 | 1 |
| ATTGGCG | 18305 | 0.0 | 12.834564 | 6 |
| TGAGTTA | 7885 | 0.0 | 12.78348 | 2 |
| CCGTAAA | 11765 | 0.0 | 12.715288 | 33 |

Produced by FastQC (version 0.11.5)
