# Supplementary material for: Multidimensional chromatin profiling of zebrafish pancreas to uncover and investigate disease-relevant enhancers
Source: Nat Commun. 2022 Apr 11;13:1945. doi: 10.1038/s41467-022-29551-7 (PMC9001708; doi:10.1038/s41467-022-29551-7)
Supplement: Supplementary file 3 — Supplementary data1-17 [file 41467_2022_29551_MOESM3_ESM.zip › SupplementaryFile1_FASTQC_reports/Supplementary data 11_RNA-seq Endocrine old fastqc 2 .html]

FCHGVKNBBXX-HKZEBggcRAAERAAPEI-207\_L3\_1.fq FastQC Report 

FastQC Report

Wed 5 Jul 2017  
FCHGVKNBBXX-HKZEBggcRAAERAAPEI-207\_L3\_1.fq

## Summary

- Basic Statistics
- Per base sequence quality
- Per tile sequence quality
- Per sequence quality scores
- Per base sequence content
- Per sequence GC content
- Per base N content
- Sequence Length Distribution
- Sequence Duplication Levels
- Overrepresented sequences
- Adapter Content
- Kmer Content

## Basic Statistics

| Measure | Value |
| --- | --- |
| Filename | FCHGVKNBBXX-HKZEBggcRAAERAAPEI-207\_L3\_1.fq |
| File type | Conventional base calls |
| Encoding | Sanger / Illumina 1.9 |
| Total Sequences | 34211212 |
| Sequences flagged as poor quality | 0 |
| Sequence length | 50 |
| %GC | 47 |

## Per base sequence quality

## Per tile sequence quality

## Per sequence quality scores

## Per base sequence content

## Per sequence GC content

## Per base N content

## Sequence Length Distribution

## Sequence Duplication Levels

## Overrepresented sequences

| Sequence | Count | Percentage | Possible Source |
| --- | --- | --- | --- |
| CTTGCCTTGACTGTAGCCCTCGTGGCATGTCAACAATTCAACCTTGTTCC | 49218 | 0.14386511650040343 | No Hit |

## Adapter Content

## Kmer Content

| Sequence | Count | PValue | Obs/Exp Max | Max Obs/Exp Position |
| --- | --- | --- | --- | --- |
| CTTGCCT | 14740 | 0.0 | 19.717379 | 1 |
| CCCTCGT | 16210 | 0.0 | 18.238668 | 17 |
| TGCCTTG | 15885 | 0.0 | 17.877794 | 3 |
| CCTCGTG | 17165 | 0.0 | 17.262379 | 18 |
| GCCCTCG | 17115 | 0.0 | 17.248545 | 16 |
| CTTGACT | 16655 | 0.0 | 17.2366 | 6 |
| TAGCCCT | 20340 | 0.0 | 17.10923 | 14 |
| TTAAGCG | 10610 | 0.0 | 16.607447 | 7 |
| GCCTTGA | 17770 | 0.0 | 16.415037 | 4 |
| TGTAGCC | 20370 | 0.0 | 16.230911 | 12 |
| GTTGTGC | 7970 | 0.0 | 15.893313 | 1 |
| TAAGCGT | 11110 | 0.0 | 15.860037 | 8 |
| CCTTGTT | 20440 | 0.0 | 15.756114 | 42 |
| TTGACTG | 15635 | 0.0 | 15.645616 | 7 |
| CTGTAGC | 21380 | 0.0 | 15.577334 | 11 |
| GTGGCAT | 15250 | 0.0 | 15.261363 | 22 |
| GATTAAG | 11005 | 0.0 | 14.971922 | 5 |
| AGCCCTC | 20050 | 0.0 | 14.953968 | 15 |
| CTTGTTC | 26835 | 0.0 | 14.944638 | 43 |
| CTAGCCT | 3075 | 0.0 | 14.879665 | 13 |

Produced by FastQC (version 0.11.5)
