# Supplementary material for: Multidimensional chromatin profiling of zebrafish pancreas to uncover and investigate disease-relevant enhancers
Source: Nat Commun. 2022 Apr 11;13:1945. doi: 10.1038/s41467-022-29551-7 (PMC9001708; doi:10.1038/s41467-022-29551-7)
Supplement: Supplementary file 3 — Supplementary data1-17 [file 41467_2022_29551_MOESM3_ESM.zip › SupplementaryFile1_FASTQC_reports/Supplementary data 12_RNA-seq Endocrine young fastqc 1 .html]

FCHGVKNBBXX-HKZEBggcRAAARAAPEI-201\_L3\_1.fq FastQC Report 

FastQC Report

Wed 5 Jul 2017  
FCHGVKNBBXX-HKZEBggcRAAARAAPEI-201\_L3\_1.fq

## Summary

- Basic Statistics
- Per base sequence quality
- Per tile sequence quality
- Per sequence quality scores
- Per base sequence content
- Per sequence GC content
- Per base N content
- Sequence Length Distribution
- Sequence Duplication Levels
- Overrepresented sequences
- Adapter Content
- Kmer Content

## Basic Statistics

| Measure | Value |
| --- | --- |
| Filename | FCHGVKNBBXX-HKZEBggcRAAARAAPEI-201\_L3\_1.fq |
| File type | Conventional base calls |
| Encoding | Sanger / Illumina 1.9 |
| Total Sequences | 26623689 |
| Sequences flagged as poor quality | 0 |
| Sequence length | 50 |
| %GC | 47 |

## Per base sequence quality

## Per tile sequence quality

## Per sequence quality scores

## Per base sequence content

## Per sequence GC content

## Per base N content

## Sequence Length Distribution

## Sequence Duplication Levels

## Overrepresented sequences

| Sequence | Count | Percentage | Possible Source |
| --- | --- | --- | --- |
| CACCATTCCTCGCCTCTGCTTCGAGAACAGTGTGACCATGGCAGTGTGGC | 100704 | 0.3782496107132261 | No Hit |
| ACCATATCCACCATTCCTCGCCTCTGCTTCGAGAACAGTGTGACCATGGC | 65320 | 0.24534541400329607 | No Hit |
| CCTCGCCTCTGCTTCGAGAACAGTGTGACCATGGCAGTGTGGCTTCAGGC | 62444 | 0.2345430041644492 | No Hit |
| CTCGCCTCTGCTTCGAGAACAGTGTGACCATGGCAGTGTGGCTTCAGGCT | 62024 | 0.23296546169841453 | No Hit |
| ATCCACCATTCCTCGCCTCTGCTTCGAGAACAGTGTGACCATGGCAGTGT | 57613 | 0.21639750975155997 | No Hit |
| CTTCAGGCTGGTGCTCTGTTGGTCCTGTTGGTCGTGTCCAGTGTAAGCAC | 43376 | 0.16292257620647538 | No Hit |
| CCACCATTCCTCGCCTCTGCTTCGAGAACAGTGTGACCATGGCAGTGTGG | 39184 | 0.14717719997405318 | No Hit |
| CCATTCCTCGCCTCTGCTTCGAGAACAGTGTGACCATGGCAGTGTGGCTT | 34250 | 0.12864483205163643 | No Hit |
| CTGGTCGATGCCCTTTATCTGGTCTGTGGCCCAACAGGCTTCTTCTACAA | 32779 | 0.12311967736702455 | No Hit |
| CTGGCATTGGCGGTGGGCAAATCTCTTCAGTTGCAGTAGTTCTGCAGCTC | 32538 | 0.12221446847579989 | No Hit |
| CTTCCTTATCAGCTCGGCATGATCTTTAAATGCAAAGTCAGCCACCTCAG | 32139 | 0.12071580313306694 | No Hit |
| CTGGCATCGGCGGTGGGCAAATCTCTTCAGTTACAGTAGTTCTGCAGCTC | 31527 | 0.11841709839684501 | No Hit |
| CTCAGTTTCCTGGGCAGATTTAGGAGGAAGGAAACCCAGAAGGGGCTCAA | 29608 | 0.11120923174846281 | No Hit |
| CGTTGAGCCCCTTCTGGGTTTCCTTCCTCCTAAATCTGCCCAGGAAACTG | 28821 | 0.10825321765139309 | No Hit |
| CTTTATCTGGTCTGTGGCCCAACAGGCTTCTTCTACAACCCCAAGAGAGA | 27363 | 0.10277689166215848 | No Hit |
| GAAGAAGCCTGTTGGGCCACAGACCAGATAAAGGGCATCGACCAGATGAG | 27139 | 0.10193553568027332 | No Hit |
| CTCAACGTCTCTCTTGGGGTTGTAGAAGAAGCCTGTTGGGCCACAGACCA | 26886 | 0.10098525414716196 | No Hit |

## Adapter Content

## Kmer Content

| Sequence | Count | PValue | Obs/Exp Max | Max Obs/Exp Position |
| --- | --- | --- | --- | --- |
| ACCATAT | 17510 | 0.0 | 19.291914 | 1 |
| CCATATC | 19710 | 0.0 | 17.720703 | 2 |
| CATATCC | 19645 | 0.0 | 17.714666 | 3 |
| ACGATCC | 4090 | 0.0 | 17.479885 | 3 |
| ACGCGTA | 3670 | 0.0 | 17.442532 | 22 |
| TATCCAC | 21015 | 0.0 | 16.64356 | 5 |
| AGACGAT | 4450 | 0.0 | 16.362144 | 1 |
| ATATCCA | 21955 | 0.0 | 16.271631 | 4 |
| CGATCCA | 5330 | 0.0 | 16.054653 | 4 |
| CACGCGT | 4210 | 0.0 | 15.257527 | 21 |
| CGGGGTT | 3995 | 0.0 | 15.132827 | 1 |
| GAGTTAA | 8940 | 0.0 | 15.00966 | 3 |
| ATTGGCG | 18090 | 0.0 | 14.701656 | 6 |
| CTTACCC | 8060 | 0.0 | 14.481279 | 1 |
| TGAGTTA | 9760 | 0.0 | 13.913167 | 2 |
| CCACGCG | 4570 | 0.0 | 13.911214 | 20 |
| CTGGCAT | 33485 | 0.0 | 13.896729 | 1 |
| CTGAGTT | 11380 | 0.0 | 13.804606 | 1 |
| GGTGGGC | 31495 | 0.0 | 13.54998 | 12 |
| CGGTGGG | 31860 | 0.0 | 13.367128 | 11 |

Produced by FastQC (version 0.11.5)
