# Supplementary material for: Multidimensional chromatin profiling of zebrafish pancreas to uncover and investigate disease-relevant enhancers
Source: Nat Commun. 2022 Apr 11;13:1945. doi: 10.1038/s41467-022-29551-7 (PMC9001708; doi:10.1038/s41467-022-29551-7)
Supplement: Supplementary file 3 — Supplementary data1-17 [file 41467_2022_29551_MOESM3_ESM.zip › SupplementaryFile1_FASTQC_reports/Supplementary data 13_RNA-seq Endocrine young fastqc 2 .html]

FCHGVKNBBXX-HKZEBggcRAABRAAPEI-202\_L3\_1.fq FastQC Report 

FastQC Report

Wed 5 Jul 2017  
FCHGVKNBBXX-HKZEBggcRAABRAAPEI-202\_L3\_1.fq

## Summary

- Basic Statistics
- Per base sequence quality
- Per tile sequence quality
- Per sequence quality scores
- Per base sequence content
- Per sequence GC content
- Per base N content
- Sequence Length Distribution
- Sequence Duplication Levels
- Overrepresented sequences
- Adapter Content
- Kmer Content

## Basic Statistics

| Measure | Value |
| --- | --- |
| Filename | FCHGVKNBBXX-HKZEBggcRAABRAAPEI-202\_L3\_1.fq |
| File type | Conventional base calls |
| Encoding | Sanger / Illumina 1.9 |
| Total Sequences | 36926312 |
| Sequences flagged as poor quality | 0 |
| Sequence length | 50 |
| %GC | 48 |

## Per base sequence quality

## Per tile sequence quality

## Per sequence quality scores

## Per base sequence content

## Per sequence GC content

## Per base N content

## Sequence Length Distribution

## Sequence Duplication Levels

## Overrepresented sequences

No overrepresented sequences

## Adapter Content

## Kmer Content

| Sequence | Count | PValue | Obs/Exp Max | Max Obs/Exp Position |
| --- | --- | --- | --- | --- |
| GAATCGG | 5445 | 0.0 | 18.260664 | 4 |
| AATCGGT | 5485 | 0.0 | 17.84676 | 5 |
| TTACCCG | 6570 | 0.0 | 17.217669 | 2 |
| TACCCGA | 6605 | 0.0 | 16.985283 | 3 |
| AGAATCG | 5835 | 0.0 | 16.927036 | 3 |
| ACGCGTA | 4410 | 0.0 | 16.560713 | 22 |
| CTTACCC | 8225 | 0.0 | 16.392086 | 1 |
| CCCGACG | 7735 | 0.0 | 14.503936 | 5 |
| CACGCGT | 5260 | 0.0 | 13.926411 | 21 |
| CCACGCG | 5370 | 0.0 | 13.682104 | 20 |
| ACGATCC | 3995 | 0.0 | 13.325208 | 3 |
| CGCGTAT | 6140 | 0.0 | 13.117569 | 1 |
| CGGCGAT | 5260 | 0.0 | 12.920932 | 1 |
| ATCGGTT | 8060 | 0.0 | 12.800114 | 6 |
| CCGACGC | 8880 | 0.0 | 12.732866 | 6 |
| TCGGTTT | 8285 | 0.0 | 12.186982 | 7 |
| TGCCGTA | 6450 | 0.0 | 11.766926 | 31 |
| CTCGGAT | 7530 | 0.0 | 11.458049 | 1 |
| GCGTATC | 7225 | 0.0 | 11.33135 | 2 |
| CGACAAT | 4800 | 0.0 | 11.308963 | 1 |

Produced by FastQC (version 0.11.5)
