# Supplementary material for: Multidimensional chromatin profiling of zebrafish pancreas to uncover and investigate disease-relevant enhancers
Source: Nat Commun. 2022 Apr 11;13:1945. doi: 10.1038/s41467-022-29551-7 (PMC9001708; doi:10.1038/s41467-022-29551-7)
Supplement: Supplementary file 3 — Supplementary data1-17 [file 41467_2022_29551_MOESM3_ESM.zip › SupplementaryFile1_FASTQC_reports/Supplementary data 14_RNA-seq Exocrine old fastqc .html]

FCHGVKNBBXX-HKZEBggcRAAFRAAPEI-208\_L3\_1.fq FastQC Report 

FastQC Report

Wed 5 Jul 2017  
FCHGVKNBBXX-HKZEBggcRAAFRAAPEI-208\_L3\_1.fq

## Summary

- Basic Statistics
- Per base sequence quality
- Per tile sequence quality
- Per sequence quality scores
- Per base sequence content
- Per sequence GC content
- Per base N content
- Sequence Length Distribution
- Sequence Duplication Levels
- Overrepresented sequences
- Adapter Content
- Kmer Content

## Basic Statistics

| Measure | Value |
| --- | --- |
| Filename | FCHGVKNBBXX-HKZEBggcRAAFRAAPEI-208\_L3\_1.fq |
| File type | Conventional base calls |
| Encoding | Sanger / Illumina 1.9 |
| Total Sequences | 34313565 |
| Sequences flagged as poor quality | 0 |
| Sequence length | 50 |
| %GC | 49 |

## Per base sequence quality

## Per tile sequence quality

## Per sequence quality scores

## Per base sequence content

## Per sequence GC content

## Per base N content

## Sequence Length Distribution

## Sequence Duplication Levels

## Overrepresented sequences

| Sequence | Count | Percentage | Possible Source |
| --- | --- | --- | --- |
| CTTTGGTGTTCCTGGTGCTCCTTGGAGCTGCCTTTGCTCTGGATGATGAC | 46478 | 0.1354508049513363 | No Hit |

## Adapter Content

## Kmer Content

| Sequence | Count | PValue | Obs/Exp Max | Max Obs/Exp Position |
| --- | --- | --- | --- | --- |
| GAATCGG | 6680 | 0.0 | 19.132658 | 4 |
| AATCGGT | 7350 | 0.0 | 17.598093 | 5 |
| AGAATCG | 7460 | 0.0 | 17.132141 | 3 |
| CGTACTA | 1620 | 0.0 | 14.258691 | 35 |
| TCGGTTT | 9135 | 0.0 | 14.014902 | 7 |
| ACAATCG | 2055 | 0.0 | 13.701638 | 3 |
| CCGGGAT | 1615 | 0.0 | 13.66462 | 1 |
| GTTCAAT | 12290 | 0.0 | 13.197922 | 1 |
| CGGCGAT | 6935 | 0.0 | 12.983261 | 1 |
| TTAAGCG | 3090 | 0.0 | 12.956524 | 7 |
| TCGCTAG | 875 | 0.0 | 12.82143 | 3 |
| TGCCGTA | 8995 | 0.0 | 12.815163 | 31 |
| ATCGGTT | 10045 | 0.0 | 12.591966 | 6 |
| CGGTTTT | 10135 | 0.0 | 12.58867 | 8 |
| CAAGAAT | 12180 | 0.0 | 12.556137 | 1 |
| GATATAT | 5965 | 0.0 | 12.354068 | 5 |
| CGCCCAT | 2475 | 0.0 | 12.304783 | 1 |
| CAATCGG | 1555 | 0.0 | 12.165885 | 4 |
| CCTAAGG | 2120 | 0.0 | 12.036379 | 14 |
| CCGGTAT | 1000 | 0.0 | 11.916915 | 1 |

Produced by FastQC (version 0.11.5)
