# Supplementary material for: Multidimensional chromatin profiling of zebrafish pancreas to uncover and investigate disease-relevant enhancers
Source: Nat Commun. 2022 Apr 11;13:1945. doi: 10.1038/s41467-022-29551-7 (PMC9001708; doi:10.1038/s41467-022-29551-7)
Supplement: Supplementary file 3 — Supplementary data1-17 [file 41467_2022_29551_MOESM3_ESM.zip › SupplementaryFile1_FASTQC_reports/Supplementary data 15_RNA-seq Exocrine young fastqc .html]

FCHGVKNBBXX-HKZEBggcRAACRAAPEI-205\_L3\_1.fq FastQC Report 

FastQC Report

Wed 5 Jul 2017  
FCHGVKNBBXX-HKZEBggcRAACRAAPEI-205\_L3\_1.fq

## Summary

- Basic Statistics
- Per base sequence quality
- Per tile sequence quality
- Per sequence quality scores
- Per base sequence content
- Per sequence GC content
- Per base N content
- Sequence Length Distribution
- Sequence Duplication Levels
- Overrepresented sequences
- Adapter Content
- Kmer Content

## Basic Statistics

| Measure | Value |
| --- | --- |
| Filename | FCHGVKNBBXX-HKZEBggcRAACRAAPEI-205\_L3\_1.fq |
| File type | Conventional base calls |
| Encoding | Sanger / Illumina 1.9 |
| Total Sequences | 35021410 |
| Sequences flagged as poor quality | 0 |
| Sequence length | 50 |
| %GC | 50 |

## Per base sequence quality

## Per tile sequence quality

## Per sequence quality scores

## Per base sequence content

## Per sequence GC content

## Per base N content

## Sequence Length Distribution

## Sequence Duplication Levels

## Overrepresented sequences

| Sequence | Count | Percentage | Possible Source |
| --- | --- | --- | --- |
| CTTTGGTGTTCCTGGTGCTCCTTGGAGCTGCCTTTGCTCTGGATGATGAC | 130196 | 0.37176115981623814 | No Hit |
| CAAGAATCGGTTTTAGTTGTTTCTCATGGTGTCAGCGATCCACTGGCTGA | 56458 | 0.16120995699487828 | No Hit |
| GTTCAATCAGCAGTCATGAGGTCTTTGGTGTTCCTGGTGCTCCTTGGAGC | 44482 | 0.12701373245680284 | No Hit |
| CAAACATGCTGAGGATCCTGTTGTTGAGTGTGCTGGCCGCTTTGGCCCTG | 41505 | 0.11851321805718275 | No Hit |
| CTTGGAGCTGCCTTTGCTCTGGATGATGACAAGATTGTTGGTGGATATGA | 37006 | 0.10566679068604035 | No Hit |

## Adapter Content

## Kmer Content

| Sequence | Count | PValue | Obs/Exp Max | Max Obs/Exp Position |
| --- | --- | --- | --- | --- |
| GTTCAAT | 7920 | 0.0 | 26.858582 | 1 |
| GAATCGG | 9810 | 0.0 | 26.325533 | 4 |
| AATCGGT | 10370 | 0.0 | 24.967539 | 5 |
| AGAATCG | 10390 | 0.0 | 24.940365 | 3 |
| ATCGGTT | 11995 | 0.0 | 21.731821 | 6 |
| TTCAATC | 10555 | 0.0 | 21.643341 | 2 |
| CGGTTTT | 13055 | 0.0 | 19.866207 | 8 |
| TCGGTTT | 13780 | 0.0 | 19.06045 | 7 |
| ATCGGAT | 1615 | 0.0 | 16.889904 | 6 |
| GGGATTG | 4330 | 0.0 | 16.764828 | 11 |
| TGCCGTA | 15410 | 0.0 | 16.702534 | 31 |
| CAAGAAT | 17840 | 0.0 | 16.376623 | 1 |
| CGGATAG | 1575 | 0.0 | 16.06184 | 8 |
| ACAATCG | 2000 | 0.0 | 15.948178 | 3 |
| CCGTAAA | 16300 | 0.0 | 15.858284 | 33 |
| CAATCAG | 14575 | 0.0 | 15.8323345 | 4 |
| AATCGGA | 1845 | 0.0 | 15.61899 | 5 |
| TCGTGTA | 4940 | 0.0 | 15.592877 | 2 |
| TCAATCA | 16220 | 0.0 | 15.528436 | 3 |
| AAGAATC | 19015 | 0.0 | 15.474629 | 2 |

Produced by FastQC (version 0.11.5)
