# Supplementary material for: Multidimensional chromatin profiling of zebrafish pancreas to uncover and investigate disease-relevant enhancers
Source: Nat Commun. 2022 Apr 11;13:1945. doi: 10.1038/s41467-022-29551-7 (PMC9001708; doi:10.1038/s41467-022-29551-7)
Supplement: Supplementary file 3 — Supplementary data1-17 [file 41467_2022_29551_MOESM3_ESM.zip › SupplementaryFile1_FASTQC_reports/Supplementary data 16_RNA-seq Muscle young fastqc .html]

FCHGVKNBBXX-HKZEBggcRAAGRAAPEI-209\_L3\_1.fq FastQC Report 

FastQC Report

Wed 5 Jul 2017  
FCHGVKNBBXX-HKZEBggcRAAGRAAPEI-209\_L3\_1.fq

## Summary

- Basic Statistics
- Per base sequence quality
- Per tile sequence quality
- Per sequence quality scores
- Per base sequence content
- Per sequence GC content
- Per base N content
- Sequence Length Distribution
- Sequence Duplication Levels
- Overrepresented sequences
- Adapter Content
- Kmer Content

## Basic Statistics

| Measure | Value |
| --- | --- |
| Filename | FCHGVKNBBXX-HKZEBggcRAAGRAAPEI-209\_L3\_1.fq |
| File type | Conventional base calls |
| Encoding | Sanger / Illumina 1.9 |
| Total Sequences | 35907057 |
| Sequences flagged as poor quality | 0 |
| Sequence length | 50 |
| %GC | 48 |

## Per base sequence quality

## Per tile sequence quality

## Per sequence quality scores

## Per base sequence content

## Per sequence GC content

## Per base N content

## Sequence Length Distribution

## Sequence Duplication Levels

## Overrepresented sequences

No overrepresented sequences

## Adapter Content

## Kmer Content

| Sequence | Count | PValue | Obs/Exp Max | Max Obs/Exp Position |
| --- | --- | --- | --- | --- |
| TAAGGCG | 2185 | 0.0 | 21.94741 | 20 |
| TCGATCG | 1465 | 0.0 | 20.12078 | 3 |
| CGATCGA | 1470 | 0.0 | 19.603573 | 4 |
| CTACCGA | 2570 | 0.0 | 16.348568 | 23 |
| TCTAAGG | 3105 | 0.0 | 16.08209 | 18 |
| CCGCGTA | 1650 | 0.0 | 15.731899 | 27 |
| CACTCGA | 4340 | 0.0 | 15.256609 | 4 |
| ACTCGAC | 3905 | 0.0 | 15.209818 | 5 |
| GCGAGTG | 3355 | 0.0 | 14.621496 | 24 |
| CGCCTAC | 5580 | 0.0 | 14.586321 | 17 |
| TCGCGTA | 710 | 0.0 | 14.562053 | 28 |
| CCGCCTA | 5610 | 0.0 | 14.547532 | 16 |
| CGTACTA | 2115 | 0.0 | 14.458217 | 35 |
| CCACTCG | 4485 | 0.0 | 14.223721 | 3 |
| CCGAGCG | 10595 | 0.0 | 14.119348 | 38 |
| GGTAGGT | 6550 | 0.0 | 14.038318 | 10 |
| CTCAGAT | 19480 | 0.0 | 13.750256 | 1 |
| CGAGCGC | 10905 | 0.0 | 13.73803 | 39 |
| CTCGCAT | 5320 | 0.0 | 13.644742 | 1 |
| GCGCACC | 10950 | 0.0 | 13.621302 | 42 |

Produced by FastQC (version 0.11.5)
