# Supplementary material for: Multidimensional chromatin profiling of zebrafish pancreas to uncover and investigate disease-relevant enhancers
Source: Nat Commun. 2022 Apr 11;13:1945. doi: 10.1038/s41467-022-29551-7 (PMC9001708; doi:10.1038/s41467-022-29551-7)
Supplement: Supplementary file 3 — Supplementary data1-17 [file 41467_2022_29551_MOESM3_ESM.zip › SupplementaryFile1_FASTQC_reports/Supplementary data 17_RNA-seq Muscle old fastqc .html]

FCHGVKNBBXX-HKZEBggcRAAHRAAPEI-210\_L3\_1.fq FastQC Report 

FastQC Report

Wed 5 Jul 2017  
FCHGVKNBBXX-HKZEBggcRAAHRAAPEI-210\_L3\_1.fq

## Summary

- Basic Statistics
- Per base sequence quality
- Per tile sequence quality
- Per sequence quality scores
- Per base sequence content
- Per sequence GC content
- Per base N content
- Sequence Length Distribution
- Sequence Duplication Levels
- Overrepresented sequences
- Adapter Content
- Kmer Content

## Basic Statistics

| Measure | Value |
| --- | --- |
| Filename | FCHGVKNBBXX-HKZEBggcRAAHRAAPEI-210\_L3\_1.fq |
| File type | Conventional base calls |
| Encoding | Sanger / Illumina 1.9 |
| Total Sequences | 37737419 |
| Sequences flagged as poor quality | 0 |
| Sequence length | 50 |
| %GC | 48 |

## Per base sequence quality

## Per tile sequence quality

## Per sequence quality scores

## Per base sequence content

## Per sequence GC content

## Per base N content

## Sequence Length Distribution

## Sequence Duplication Levels

## Overrepresented sequences

No overrepresented sequences

## Adapter Content

## Kmer Content

| Sequence | Count | PValue | Obs/Exp Max | Max Obs/Exp Position |
| --- | --- | --- | --- | --- |
| TCGATCG | 1825 | 0.0 | 16.513334 | 3 |
| CGATCGA | 1670 | 0.0 | 16.333786 | 4 |
| CCGCGTA | 2670 | 0.0 | 15.983721 | 27 |
| CGGAATC | 3615 | 0.0 | 14.489485 | 2 |
| CGTACTA | 2920 | 0.0 | 14.465135 | 35 |
| TCGCGTA | 1240 | 0.0 | 14.36982 | 28 |
| CCGAGCG | 11190 | 0.0 | 13.859666 | 38 |
| CGAGCGC | 11465 | 0.0 | 13.431166 | 39 |
| GCGCACC | 11800 | 0.0 | 13.1806 | 42 |
| ACTCGAC | 3620 | 0.0 | 13.125832 | 5 |
| CCGCCTA | 4485 | 0.0 | 12.997589 | 16 |
| CTACCGA | 2260 | 0.0 | 12.848344 | 23 |
| CGCACCT | 12025 | 0.0 | 12.824638 | 43 |
| CGCGTAC | 3385 | 0.0 | 12.60756 | 28 |
| GTACCGG | 8365 | 0.0 | 12.59719 | 35 |
| AACTACG | 5520 | 0.0 | 12.593014 | 10 |
| GTCGCGT | 1420 | 0.0 | 12.548276 | 27 |
| CTCAGAT | 17305 | 0.0 | 12.458818 | 1 |
| CAATCGA | 2930 | 0.0 | 12.387909 | 4 |
| ACCGAGC | 12665 | 0.0 | 12.3323965 | 37 |

Produced by FastQC (version 0.11.5)
