# Supplementary material for: Multidimensional chromatin profiling of zebrafish pancreas to uncover and investigate disease-relevant enhancers
Source: Nat Commun. 2022 Apr 11;13:1945. doi: 10.1038/s41467-022-29551-7 (PMC9001708; doi:10.1038/s41467-022-29551-7)
Supplement: Supplementary file 3 — Supplementary data1-17 [file 41467_2022_29551_MOESM3_ESM.zip › SupplementaryFile1_FASTQC_reports/Supplementary data 2_Pancreas H3K27ac ChIP-seq fastqc 2 .html]

chip2.fastq FastQC Report 

FastQC Report

quarta 20 nov 2019  
chip2.fastq

## Summary

- Basic Statistics
- Per base sequence quality
- Per tile sequence quality
- Per sequence quality scores
- Per base sequence content
- Per sequence GC content
- Per base N content
- Sequence Length Distribution
- Sequence Duplication Levels
- Overrepresented sequences
- Adapter Content
- Kmer Content

## Basic Statistics

| Measure | Value |
| --- | --- |
| Filename | chip2.fastq |
| File type | Conventional base calls |
| Encoding | Sanger / Illumina 1.9 |
| Total Sequences | 27406818 |
| Sequences flagged as poor quality | 0 |
| Sequence length | 49 |
| %GC | 39 |

## Per base sequence quality

## Per tile sequence quality

## Per sequence quality scores

## Per base sequence content

## Per sequence GC content

## Per base N content

## Sequence Length Distribution

## Sequence Duplication Levels

## Overrepresented sequences

No overrepresented sequences

## Adapter Content

## Kmer Content

| Sequence | Count | PValue | Obs/Exp Max | Max Obs/Exp Position |
| --- | --- | --- | --- | --- |
| TAGATCG | 9255 | 0.0 | 35.680958 | 43 |
| GAGATCG | 12680 | 0.0 | 35.249825 | 43 |
| CAGATCG | 11440 | 0.0 | 33.52668 | 43 |
| AAGATCG | 4460 | 0.0 | 25.548342 | 43 |
| CTAGATC | 8140 | 0.0 | 15.081115 | 43 |
| AGAGATC | 14065 | 0.0 | 12.671747 | 42 |
| TGAGATC | 14485 | 0.0 | 11.7254715 | 42 |
| ATAGATC | 8750 | 0.0 | 11.572698 | 42 |
| ACAGATC | 15795 | 0.0 | 10.984382 | 42 |
| GGAGATC | 8710 | 0.0 | 10.04611 | 42 |
| TTAGATC | 9370 | 0.0 | 9.131986 | 42 |
| CCAGATC | 12325 | 0.0 | 8.791548 | 43 |
| CGAGATC | 3620 | 0.0 | 8.433376 | 43 |
| GTAGATC | 5745 | 0.0 | 7.9335504 | 43 |
| CAAGATC | 7575 | 0.0 | 7.918507 | 43 |
| CCTAGAT | 4620 | 0.0 | 7.8178806 | 43 |
| TCAGATC | 12690 | 0.0 | 6.963089 | 43 |
| GCTGGCT | 11555 | 0.0 | 6.6060634 | 3 |
| TGCTGGC | 11950 | 0.0 | 6.497503 | 2 |
| TCAACGT | 9150 | 0.0 | 6.297025 | 25 |

Produced by FastQC (version 0.11.5)
