# Supplementary material for: Multidimensional chromatin profiling of zebrafish pancreas to uncover and investigate disease-relevant enhancers
Source: Nat Commun. 2022 Apr 11;13:1945. doi: 10.1038/s41467-022-29551-7 (PMC9001708; doi:10.1038/s41467-022-29551-7)
Supplement: Supplementary file 3 — Supplementary data1-17 [file 41467_2022_29551_MOESM3_ESM.zip › SupplementaryFile1_FASTQC_reports/Supplementary data 3_4C-seq Arid1ab fastqc 1.html]

Arid1a\_1\_Arid1ab.fastq.gz FastQC Report 

FastQC Report

Thu 26 Oct 2017  
Arid1a\_1\_Arid1ab.fastq.gz

## Summary

- Basic Statistics
- Per base sequence quality
- Per tile sequence quality
- Per sequence quality scores
- Per base sequence content
- Per sequence GC content
- Per base N content
- Sequence Length Distribution
- Sequence Duplication Levels
- Overrepresented sequences
- Adapter Content
- Kmer Content

## Basic Statistics

| Measure | Value |
| --- | --- |
| Filename | Arid1a\_1\_Arid1ab.fastq.gz |
| File type | Conventional base calls |
| Encoding | Sanger / Illumina 1.9 |
| Total Sequences | 4417017 |
| Sequences flagged as poor quality | 0 |
| Sequence length | 29 |
| %GC | 41 |

## Per base sequence quality

## Per tile sequence quality

## Per sequence quality scores

## Per base sequence content

## Per sequence GC content

## Per base N content

## Sequence Length Distribution

## Sequence Duplication Levels

## Overrepresented sequences

| Sequence | Count | Percentage | Possible Source |
| --- | --- | --- | --- |
| AATGCCAAAGAAAGAACTCTCATTTCTGA | 106892 | 2.4200042698499917 | No Hit |
| TTCTTCACAGCGGTCGAGTGAATGATGTC | 84484 | 1.9126935667216134 | No Hit |
| TATTTGCAATTCACAGGGCAGCAGTAGTC | 52449 | 1.18743034043111 | No Hit |
| TTAACAGGGGGCAGCAGTAGTCAGTATGT | 49144 | 1.1126060868681285 | No Hit |
| CACCTGCACTAAATCCTGGCTGAGACTGG | 48927 | 1.1076932690093788 | No Hit |
| CTTCACTGACCTCAATCAGATTTTCAAAA | 46308 | 1.0483998589998635 | No Hit |
| TTAAAACGAAACAAAAGTTTATTGCTACT | 35232 | 0.7976423907809276 | No Hit |
| AATGCCAAAGCAAGAACTTTCATTTCAGA | 29388 | 0.6653359042992137 | No Hit |
| TCACAAACAACTATGGGATGATAAACATC | 27985 | 0.6335723860696031 | No Hit |
| AATGCCAAAGCAAGAACTTTCATTTCTGA | 21910 | 0.49603612573825273 | No Hit |
| TTTCCGTCGATAAAAACCTGTGGCGGATT | 21769 | 0.4928439261157474 | No Hit |
| ATGTTACTTTGAAATATTTTACTATAACT | 21755 | 0.4925269701248603 | No Hit |
| TGTGAGTTCGGCTTCTCTCCGGTGGCTTG | 20885 | 0.47283041926259284 | No Hit |
| CTTGGAAGGACCAGGGCAGCAGTAGTCAG | 20803 | 0.47097396274454006 | No Hit |
| CAGAAACGAGCCTCCCAGCAGACTCAAGA | 18029 | 0.4081713971216321 | No Hit |
| CATGCGCAGTTTATTAATAAGAGTATTCA | 16685 | 0.3777436219964741 | No Hit |
| GGGTATTCCTAGCTTCTAATGAACCGTAG | 16184 | 0.36640112546544423 | No Hit |
| ATACTAAATTAGACTTGATTTAAAACAAA | 16081 | 0.3640692349610608 | No Hit |
| ACCAACATTTAGAATGAACAAGACTAAAA | 15545 | 0.3519343484528133 | No Hit |
| GGGCAGCAGTAGTCAGTATGTCGTATGCC | 14870 | 0.3366525417493299 | No Hit |
| TTTAAAATAGCAAAGGCTTTAATGTGCGT | 14520 | 0.32872864197715335 | No Hit |
| TGAGTGATAATAAATCTGAAGGGAATCTG | 13653 | 0.30910001025579026 | No Hit |
| CATTACTTGGTGTTATACATTCACGCACT | 13474 | 0.3050475015151628 | No Hit |
| TCCCAGAGGAGCTGGAGGAAGTGTCTGGG | 13217 | 0.2992290951110218 | No Hit |
| TGCAGACGGACCTCACACTCTACAAGTCA | 12827 | 0.2903996067934536 | No Hit |
| TCAGTCTTCCTTTTGTAAGAAGGATAAGT | 11667 | 0.26413753897709696 | No Hit |
| TTTTGTATTTTATATTTATATATTTTTAT | 10553 | 0.23891689798794075 | No Hit |
| TGCTTGTCTTGTTCAGACGCTTCAAACAA | 9687 | 0.21931090598021247 | No Hit |
| ACTCTCTCTCTCTCTCTCTAAAGTGCTGT | 9416 | 0.21317554358518429 | No Hit |
| TGCTCGTCTTGTTCAGCTGCTTCAAACAA | 9088 | 0.2057497175129731 | No Hit |
| TGTCTTTTTTATTAAGCTCGACGAAAATC | 8955 | 0.20273863559954605 | No Hit |
| AGACACAAACAGCTTTGGATTTGATATCC | 8709 | 0.19716926604538768 | No Hit |
| ACATCTGCTCTGTGGTGGCCTCTTTGCCC | 8693 | 0.196807030627231 | No Hit |
| CAGACGCCTCAGATGAGAGCGAGTGTTAA | 8656 | 0.19596936122274378 | No Hit |
| CGGTATGGTCCAATGAACCTGGGTGCCAA | 8172 | 0.18501173982350533 | No Hit |
| CACTCGAGTGTGCATAAATAATGATACAA | 7961 | 0.1802347602465646 | No Hit |
| GCCTTCTTCCTCGTTATTTCCTCCTTCCT | 7947 | 0.17991780425567755 | No Hit |
| TACAGCTGTAATTACACACACACACACAC | 7915 | 0.17919333341936425 | No Hit |
| AAAGTCCTGCTGCAGAGACCAGACCGTTC | 7715 | 0.1746653906924062 | No Hit |
| ACCCCTGGACTGAAGTGTTTACATTTATG | 7664 | 0.1735107652970319 | No Hit |
| AGGGCAGCAGTAGTCAGTATGTCGTATGC | 7457 | 0.16882434457463036 | No Hit |
| ATTTCAGAGCAGACGCTGAATTAGAAAGG | 7415 | 0.16787347660196916 | No Hit |
| ATCATGGGCAGCAGTAGTCAGTATGTCGT | 7317 | 0.16565478466575972 | No Hit |
| TCAGGGGCAGCAGTAGTCAGTATGTCGTA | 7311 | 0.16551894638395098 | No Hit |
| ATGCTGTATAGGTCTTTCTCTGGTCTTTC | 7296 | 0.16517935067942913 | No Hit |
| ACGTTTATATGTTCTTCTTAATGTCAACT | 7219 | 0.1634360927295503 | No Hit |
| GGTGTATGATGTGCAGCACATCTTTAAAG | 7126 | 0.1613305993615148 | No Hit |
| TGGCATGGTGTAGATCTTTCCGTCGATAA | 7062 | 0.15988165768888823 | No Hit |
| TACAGCTGTAATTACACACACACACACGC | 6878 | 0.15571595038008681 | No Hit |
| AGCGCGTGACAGTAATGGTGACCTAATAA | 6469 | 0.14645630750345764 | No Hit |
| TGTAGACAACACTAGCCTCAGTCCTAGTG | 6251 | 0.1415208499310734 | No Hit |
| AGTCAGTTTAACTTGCTGGTCAGACACAT | 6227 | 0.14097749680383842 | No Hit |
| TGCAGACTGACCTCACACTCTACAAGTCA | 6224 | 0.14090957766293408 | No Hit |
| TTAATCTGTTACTGCTAATTTCTGGCTTT | 6163 | 0.13952855513121185 | No Hit |
| ACTGACTCGTCGTGTGACCACAAACGGCC | 6122 | 0.13860032687218546 | No Hit |
| ATGGTAAATGTGTATACACCAGGTGACGT | 5934 | 0.1343440607088449 | No Hit |
| CAGTTCATGTTTTGGCGCTGAAGCATATA | 5891 | 0.13337055302254894 | No Hit |
| AATTGAAAGTAATAATTATGGGCAGCAGT | 5850 | 0.13244232476352255 | No Hit |
| TCCAGGAACAGGATTAAGCACCCCTGCTA | 5836 | 0.13212536877263548 | No Hit |
| CATTAAATTCCAACCATGACAGAGCCGTG | 5741 | 0.1299745959773304 | No Hit |
| AACTGACCCATCCTCCTCCTTCCCTAAAC | 5711 | 0.1292954045682867 | No Hit |
| TATGTGGATTTGTAACAAAAATGTATAAT | 5710 | 0.1292727648546519 | No Hit |
| TCACTAGTGCTTGATTAAGGTACAGCGGC | 5702 | 0.12909164714557358 | No Hit |
| ACACGGTTTGCTTTTGTCCTGTCAAATGA | 5660 | 0.1281407791729124 | No Hit |
| TGGGCAGCAGTAGTCAGTATGTCGTATGC | 5623 | 0.12730310976842515 | No Hit |
| GCCGGGGCAGCAGTAGTCAGTATGTCGTA | 5596 | 0.12669183750028584 | No Hit |
| TTATTGCCACAGTATATTGTCATGATAAC | 5520 | 0.12497121926404178 | No Hit |
| TTCAAACGCAGTATAGAGCATGATGAGCT | 5448 | 0.12334115988233688 | No Hit |
| TAGAACCATGGCCTAGTTCTCAGTGTGAC | 5441 | 0.12318268188689335 | No Hit |
| TCAAACTTTGAAACGGTACAGCGGCCAGG | 5391 | 0.12205069620515384 | No Hit |
| ATAGGGCAGCAGTAGTCAGTATGTCGTAT | 5336 | 0.12080551195524038 | No Hit |
| ACTAGTAAGATGTGTCATTATTTGTAACT | 5275 | 0.11942448942351819 | No Hit |
| GGATGCGTAGGGCAGCAGTAGTCAGTATG | 5184 | 0.11736427548275227 | No Hit |
| ACCCCTGATTATACACACTGTTGGAAGCT | 5183 | 0.11734163576911749 | No Hit |
| AACACCTCTGATGATAAAACAAGCAAAAT | 5150 | 0.1165945252191694 | No Hit |
| AGTGAGTTCGGCTTCATTCCGGTGGCTTG | 5108 | 0.11564365724650821 | No Hit |
| CCAGGAGTACATTATTTTTAGTCAATACT | 5069 | 0.1147607084147514 | No Hit |
| TGCTTGTCTTGTTCAGGAGCTTCAAACAT | 5023 | 0.11371928158755105 | No Hit |
| ACGATTCTGGATTGTCTTTAAAACATCTG | 5017 | 0.11358344330574231 | No Hit |
| AGTGGTGCTGCATCAGCAAGCGTCTGCAT | 4999 | 0.11317592846031609 | No Hit |
| CTAATTTTTAGGAGGGAGTATTTTAAGCC | 4946 | 0.1119760236376722 | No Hit |
| TACTGGCCATCAACTGCTACTGAGACAGG | 4938 | 0.11179490592859388 | No Hit |
| ATGTCCAGTTCAGTGGACTGGTGTGCTGG | 4919 | 0.11136475136953289 | No Hit |
| TCACTCTAACAAGACAATCCGTCATGCCA | 4894 | 0.11079875852866311 | No Hit |
| CTCATAAGTACGGCGGCCAGGAAGGAGCT | 4872 | 0.11030068482869775 | No Hit |
| TCTGCCACACACTAATGAACTTAGTTAAA | 4839 | 0.10955357427874966 | No Hit |
| AGCTTCTTACAAAACCTCGTCTGGGTACG | 4810 | 0.10889702258334075 | No Hit |
| TTGGGGCAGCAGTAGTCAGTATGTCGTAT | 4742 | 0.107357522056175 | No Hit |
| AGTGTAGGGGCAGCAGTAGTCAGTATGTC | 4694 | 0.1062708158017051 | No Hit |
| ATGCTTATAAAGATGCTTATGCTTATGTT | 4682 | 0.1059991392380876 | No Hit |
| TTCGAGTCGACACTGTTTATCTGAAATGC | 4653 | 0.10534258754267868 | No Hit |
| GGTTATTCCTAGCTTCTAATGAACCGTAG | 4607 | 0.10430116071547835 | No Hit |
| AGCTGTCAATCACTCAAGACTTTCAGATG | 4587 | 0.10384836644278254 | No Hit |
| TCGCCAGACTTCCCTTTAAAGCATGCACG | 4577 | 0.10362196930643462 | No Hit |
| ATTTAGTGAGAGAATTAGATTCAAGATGT | 4446 | 0.10065616682027713 | No Hit |

## Adapter Content

## Kmer Content

| Sequence | Count | PValue | Obs/Exp Max | Max Obs/Exp Position |
| --- | --- | --- | --- | --- |
| CAGCCCG | 25 | 0.0019927048 | 23.002401 | 19 |
| GGCGCTA | 95 | 0.0 | 23.002401 | 19 |
| ACTCCGA | 25 | 0.0019927048 | 23.002401 | 17 |
| GATCGGT | 35 | 2.2808388E-5 | 23.0024 | 17 |
| GGGTCCG | 45 | 2.6601265E-7 | 23.0024 | 17 |
| GATCCGA | 240 | 0.0 | 23.0024 | 19 |
| GTCCGGA | 120 | 0.0 | 23.0024 | 16 |
| CGCGTAC | 30 | 2.126181E-4 | 23.0024 | 16 |
| CCGCCGG | 30 | 2.1263442E-4 | 23.00214 | 15 |
| GGGTACG | 615 | 0.0 | 23.001621 | 23 |
| GGTACCA | 30 | 2.1266704E-4 | 23.00162 | 23 |
| CCCTAGC | 40 | 2.459692E-6 | 23.000837 | 7 |
| GGTACGT | 40 | 2.459934E-6 | 23.000578 | 20 |
| GGATCCT | 40 | 2.459934E-6 | 23.000578 | 3 |
| CGGATAC | 135 | 0.0 | 23.000578 | 2 |
| CGCAACG | 70 | 3.6379788E-12 | 23.000578 | 1 |
| GATCGCG | 100 | 0.0 | 23.000576 | 2 |
| CGCGCAG | 50 | 2.8911018E-8 | 23.000576 | 3 |
| GCACGAC | 25 | 0.0019936236 | 23.000576 | 9 |
| GAACGCG | 30 | 2.1273231E-4 | 23.000576 | 20 |

Produced by FastQC (version 0.11.5)
