# Supplementary material for: Multidimensional chromatin profiling of zebrafish pancreas to uncover and investigate disease-relevant enhancers
Source: Nat Commun. 2022 Apr 11;13:1945. doi: 10.1038/s41467-022-29551-7 (PMC9001708; doi:10.1038/s41467-022-29551-7)
Supplement: Supplementary file 3 — Supplementary data1-17 [file 41467_2022_29551_MOESM3_ESM.zip › SupplementaryFile1_FASTQC_reports/Supplementary data 4_4C-seq Arid1ab fastqc 2 .html]

NeoHDF\_BC0\_1\_Arid1a\_Pan3.fastq FastQC Report 

FastQC Report

Mon 6 Jan 2020  
NeoHDF\_BC0\_1\_Arid1a\_Pan3.fastq

## Summary

- Basic Statistics
- Per base sequence quality
- Per tile sequence quality
- Per sequence quality scores
- Per base sequence content
- Per sequence GC content
- Per base N content
- Sequence Length Distribution
- Sequence Duplication Levels
- Overrepresented sequences
- Adapter Content
- Kmer Content

## Basic Statistics

| Measure | Value |
| --- | --- |
| Filename | NeoHDF\_BC0\_1\_Arid1a\_Pan3.fastq |
| File type | Conventional base calls |
| Encoding | Sanger / Illumina 1.9 |
| Total Sequences | 6032797 |
| Sequences flagged as poor quality | 0 |
| Sequence length | 29 |
| %GC | 39 |

## Per base sequence quality

## Per tile sequence quality

## Per sequence quality scores

## Per base sequence content

## Per sequence GC content

## Per base N content

## Sequence Length Distribution

## Sequence Duplication Levels

## Overrepresented sequences

| Sequence | Count | Percentage | Possible Source |
| --- | --- | --- | --- |
| GATTTGATTAGAAACGAGGCTGGTTTGGG | 182442 | 3.0241693861073062 | No Hit |
| TGGTTTTATCACATTCTGAATTGAACGTT | 11763 | 0.1949841839531481 | No Hit |
| AGGCTCTGGTTATCTACTGAAATGCTGAA | 11741 | 0.194619510651527 | No Hit |
| CCCGGGCCAGAAGGAGCTGCAAGACGGGG | 9935 | 0.16468314780026577 | No Hit |
| TGCAAAGCATTCCACTCCATTACTTCTTT | 9327 | 0.15460490382819114 | No Hit |
| CTCTCTTCAATTTACTGTGTGTGCTTTCA | 8851 | 0.14671469966584325 | No Hit |
| AGCAAACAAACTAGCCTGACAGAACTAGC | 8698 | 0.14417856261365997 | No Hit |
| ATTCACAAAAACACCCTCCTCCCGGCTCT | 8398 | 0.13920574486428103 | No Hit |
| TAAAACGTTTTTTTGAAAGCTTTTAAAGT | 8242 | 0.13661987963460398 | No Hit |
| TCATTTAGGTGAATGTCTGCGGCCACGTC | 8118 | 0.13456444829819403 | No Hit |
| CAGCCATAAAATGCATCATTCTTTTTTGT | 7596 | 0.1259117454142747 | No Hit |
| ACACAGACTTCAGCAGTGAATAATGACTT | 7535 | 0.1249006058052343 | No Hit |
| ATGTGCTGAATGTTAATCCACCATCTACA | 7432 | 0.12319327171128086 | No Hit |
| GCCTCTTTCTGTAGTAGTTTTTTGATTTC | 7201 | 0.11936420204425907 | No Hit |
| GACACAATATGAAAAAATGTATATTCGTT | 6997 | 0.1159826859746814 | No Hit |
| CTGAAGACTACATCCAATGGACACCAAGC | 6884 | 0.11410959128908199 | No Hit |
| TCAGCGTTTGCAGATGAGAACAGCTCTCA | 6761 | 0.11207073601183665 | No Hit |
| CAGGTTTGCTTTGTTGTGGCCTGCAGTAA | 6695 | 0.11097671610697327 | No Hit |
| AGATGTCAGAGGCACTTCAATAATCACAC | 6592 | 0.10926938201301983 | No Hit |
| CGGATTCGCTTTTAGTCTCCTCTTAATAG | 6472 | 0.10728025491326826 | No Hit |
| GGATAAAATCCCTAGGAGGAGTTCGTTAA | 6377 | 0.10570552929263159 | No Hit |
| GGTCATCATGTCTGAAAAAAATAACTAAA | 6258 | 0.10373297825204462 | No Hit |
| TTTCATGAGACTTGAACCGGCGAACAGCA | 6182 | 0.10247319775553529 | No Hit |
| AAGCTCTGCCTATTACATCCAGAGCTGGT | 6105 | 0.10119684119986136 | No Hit |
| TGCGCGTTTTGAACCGCTGTCTGCACAAG | 6054 | 0.10035146218246693 | No Hit |

## Adapter Content

## Kmer Content

| Sequence | Count | PValue | Obs/Exp Max | Max Obs/Exp Position |
| --- | --- | --- | --- | --- |
| CGGGTCC | 70 | 3.6379788E-12 | 23.00044 | 3 |
| AGCCGCG | 65 | 3.8198777E-11 | 23.00044 | 8 |
| GATCGGG | 35 | 2.2825217E-5 | 23.00044 | 1 |
| CGACCTA | 180 | 0.0 | 23.00044 | 5 |
| GCCGGAC | 100 | 0.0 | 23.00044 | 5 |
| GACCCCG | 50 | 2.8916475E-8 | 23.00044 | 2 |
| CCGTCGG | 270 | 0.0 | 23.00044 | 3 |
| CAGGCGA | 25 | 0.0019937768 | 23.00044 | 2 |
| CGGGCGC | 35 | 2.2825217E-5 | 23.00044 | 4 |
| CGTGTCG | 135 | 0.0 | 23.00044 | 6 |
| ACGTAGC | 140 | 0.0 | 23.00044 | 1 |
| TAGTGCC | 135 | 0.0 | 23.00044 | 5 |
| GACTCAC | 70 | 3.6379788E-12 | 23.00044 | 7 |
| CCCGCCG | 90 | 0.0 | 23.00044 | 2 |
| TGCGTCG | 180 | 0.0 | 23.00044 | 7 |
| CGACGGG | 295 | 0.0 | 23.000439 | 3 |
| GCGAACT | 30 | 2.1275344E-4 | 23.000439 | 5 |
| ACCGACG | 85 | 0.0 | 23.000439 | 4 |
| CGCGCGA | 40 | 2.4603087E-6 | 23.000439 | 7 |
| CGTCTAG | 30 | 2.1275344E-4 | 23.000439 | 6 |

Produced by FastQC (version 0.11.5)
