# Supplementary material for: Multidimensional chromatin profiling of zebrafish pancreas to uncover and investigate disease-relevant enhancers
Source: Nat Commun. 2022 Apr 11;13:1945. doi: 10.1038/s41467-022-29551-7 (PMC9001708; doi:10.1038/s41467-022-29551-7)
Supplement: Supplementary file 3 — Supplementary data1-17 [file 41467_2022_29551_MOESM3_ESM.zip › SupplementaryFile1_FASTQC_reports/Supplementary data 5_4C-seq Ptf1a fastqc .html]

Arid1a\_1\_Ptf1a.fastq.gz FastQC Report 

FastQC Report

Thu 26 Oct 2017  
Arid1a\_1\_Ptf1a.fastq.gz

## Summary

- Basic Statistics
- Per base sequence quality
- Per tile sequence quality
- Per sequence quality scores
- Per base sequence content
- Per sequence GC content
- Per base N content
- Sequence Length Distribution
- Sequence Duplication Levels
- Overrepresented sequences
- Adapter Content
- Kmer Content

## Basic Statistics

| Measure | Value |
| --- | --- |
| Filename | Arid1a\_1\_Ptf1a.fastq.gz |
| File type | Conventional base calls |
| Encoding | Sanger / Illumina 1.9 |
| Total Sequences | 6949357 |
| Sequences flagged as poor quality | 0 |
| Sequence length | 27 |
| %GC | 52 |

## Per base sequence quality

## Per tile sequence quality

## Per sequence quality scores

## Per base sequence content

## Per sequence GC content

## Per base N content

## Sequence Length Distribution

## Sequence Duplication Levels

## Overrepresented sequences

| Sequence | Count | Percentage | Possible Source |
| --- | --- | --- | --- |
| AATGATACGGCGACCACCGAACACTCT | 238517 | 3.4322168223621268 | No Hit |
| CAGACAGGACATGCTACTAAATCGTAT | 215178 | 3.0963728011095126 | No Hit |
| GGTGGCCAGACAGGACATGCTACTAAA | 165517 | 2.3817599239757 | No Hit |
| GGAAGAGCCAGACAGGACATGCTACTA | 161182 | 2.3193800519961774 | No Hit |
| TGTCCCTCAGTTACAGTAAATAAACGA | 155582 | 2.2387970570514653 | No Hit |
| GGAACCAGACAGGACATGCTACTAAAT | 153988 | 2.215859683133274 | No Hit |
| GGTGCCAGACAGGACATGCTACTAAAT | 137074 | 1.9724702587591916 | No Hit |
| GGAAGAGCCCAGACAGGACATGCTACT | 114300 | 1.6447564861036785 | No Hit |
| GGAAGCCAGACAGGACATGCTACTAAA | 80612 | 1.1599922122291315 | No Hit |
| ATGATACGGCGACCACCGAACACTCTT | 76601 | 1.1022746420999814 | No Hit |
| TTTTCCTCTTGATGCTCAAGAGTTAGT | 57759 | 0.8311416437520767 | No Hit |
| GGTGACCAGACAGGACATGCTACTAAA | 54785 | 0.788346317508224 | No Hit |
| CTCTTTCCCAGACAGGACATGCTACTA | 47851 | 0.6885673019820394 | No Hit |
| GGGGCCAGACAGGACATGCTACTAAAT | 47773 | 0.6874448959810239 | No Hit |
| GGAAGACCAGACAGGACATGCTACTAA | 46648 | 0.6712563478894522 | No Hit |
| CTCTTTCCAGACAGGACATGCTACTAA | 45583 | 0.655931189029431 | No Hit |
| GGAAGAGCGTCCAGACAGGACATGCTA | 44641 | 0.6423759780940884 | No Hit |
| GGTGTCCAGACAGGACATGCTACTAAA | 43923 | 0.6320440869565342 | No Hit |
| GGAAGAGCGCCAGACAGGACATGCTAC | 42515 | 0.6117832196561495 | No Hit |
| GGAAGAGCGTCCCAGACAGGACATGCT | 40145 | 0.5776793450099053 | No Hit |
| AGTTACAGCAGGAGAAAAATTACCTGA | 37082 | 0.5336033247392529 | No Hit |
| AATGATACGGCGACCACCGAACACTCC | 35082 | 0.5048236836875699 | No Hit |
| GGTGGTCCAGACAGGACATGCTACTAA | 34003 | 0.48929706734018696 | No Hit |
| GGGTCCAGACAGGACATGCTACTAAAT | 29384 | 0.4228304863313253 | No Hit |
| AAAACCGGCTATTTACTGATGTCATTT | 27920 | 0.4017637890814934 | No Hit |
| GGACCAGACAGGACATGCTACTAAATC | 27786 | 0.3998355531310307 | No Hit |
| GGTGGTACCAGACAGGACATGCTACTA | 26590 | 0.38262532778212427 | No Hit |
| AACACTCTTTCCCAGACAGGACATGCT | 23747 | 0.341715068027157 | No Hit |
| GGTGGTCCCAGACAGGACATGCTACTA | 22801 | 0.32810229780971106 | No Hit |
| GGGTACCAGACAGGACATGCTACTAAA | 22465 | 0.3232673181130283 | No Hit |
| GGTGGGCCAGACAGGACATGCTACTAA | 22130 | 0.3184467282368714 | No Hit |
| GGTGGTGTCCAGACAGGACATGCTACT | 20426 | 0.2939264740608376 | No Hit |
| TAGCCGTTCATACATTAGACTCAACAA | 19361 | 0.27860131520081644 | No Hit |
| CTTCCTCCGCTGCTTTTCATTAGGCAC | 19277 | 0.27739257027664577 | No Hit |
| GGTGGACCAGACAGGACATGCTACTAA | 17702 | 0.25472860294844546 | No Hit |
| GGTGTGCCAGACAGGACATGCTACTAA | 17172 | 0.24710199806974947 | No Hit |
| GGTGGTCGCCAGACAGGACATGCTACT | 16995 | 0.24455499983667556 | No Hit |
| TCAGCTGGCGGATCCCAGACAGGACAT | 16194 | 0.23302875359547653 | No Hit |
| GGAGGCCAGACAGGACATGCTACTAAA | 16082 | 0.2314170936965823 | No Hit |
| GGCCAGACAGGACATGCTACTAAATCG | 15526 | 0.22341635348421446 | No Hit |
| GTACGGCGACCACCGAACACTCTTTCC | 14958 | 0.2152429354255365 | No Hit |
| GGGCGCCAGACAGGACATGCTACTAAA | 14864 | 0.21389029229610743 | No Hit |
| GGGGGCCAGACAGGACATGCTACTAAA | 14859 | 0.2138183431934782 | No Hit |
| GTGGCCAGACAGGACATGCTACTAAAT | 14835 | 0.213472987500858 | No Hit |
| GTGGTCCAGACAGGACATGCTACTAAA | 14384 | 0.2069831784437035 | No Hit |
| GGGTGCCAGACAGGACATGCTACTAAA | 14315 | 0.20599028082742044 | No Hit |
| GGGCCGCCAGACAGGACATGCTACTAA | 13930 | 0.20045019992497148 | No Hit |
| GGGGTCCAGACAGGACATGCTACTAAA | 13908 | 0.20013362387340297 | No Hit |
| GGGTGACCAGACAGGACATGCTACTAA | 13866 | 0.1995292514113176 | No Hit |
| GGTGGTCGCCCAGACAGGACATGCTAC | 13807 | 0.198680252000293 | No Hit |
| GGTGCCGCCAGACAGGACATGCTACTA | 13465 | 0.1937589333804552 | No Hit |
| GGTGGATCCAGACAGGACATGCTACTA | 13268 | 0.19092413873686445 | No Hit |
| GGTACCAGACAGGACATGCTACTAAAT | 13194 | 0.18985929201795218 | No Hit |
| AACACTCTTTCCAGACAGGACATGCTA | 13102 | 0.18853542852957475 | No Hit |
| GGTGGGTCACCAGACAGGACATGCTAC | 12915 | 0.1858445320912424 | No Hit |
| GTGGACCAGACAGGACATGCTACTAAA | 12492 | 0.17975763800881145 | No Hit |
| GGTGGGTACCAGACAGGACATGCTACT | 12455 | 0.1792252146493553 | No Hit |
| GGTGGCCGCCAGACAGGACATGCTACT | 12335 | 0.17749843618625435 | No Hit |
| GGGCCAGACAGGACATGCTACTAAATC | 12001 | 0.17269223613062332 | No Hit |
| GGGGACCAGACAGGACATGCTACTAAA | 12000 | 0.17267784631009747 | No Hit |
| GGTGGTGGCCAGACAGGACATGCTACT | 11997 | 0.17263467684851994 | No Hit |
| GACCACCGAACACTCTTTCCAGACAGG | 11971 | 0.17226054151484807 | No Hit |
| GGTGCGCCAGACAGGACATGCTACTAA | 11822 | 0.17011645825649768 | No Hit |
| GGTCCAGACAGGACATGCTACTAAATC | 11817 | 0.17004450915386848 | No Hit |
| GGGGCCGCCAGACAGGACATGCTACTA | 11739 | 0.16892210315285286 | No Hit |
| GGGGCGTACCAGACAGGACATGCTACT | 11568 | 0.16646144384293396 | No Hit |
| GGTTGCCAGACAGGACATGCTACTAAA | 11471 | 0.16506563125192733 | No Hit |
| GGGTCGCCAGACAGGACATGCTACTAA | 11360 | 0.16346836117355892 | No Hit |
| ACCACCGAACACTCTTTCCCAGACAGG | 11315 | 0.16282081924989608 | No Hit |
| GTCACCAGACAGGACATGCTACTAAAT | 11229 | 0.1615832946846737 | No Hit |
| GTGCGCCAGACAGGACATGCTACTAAA | 11057 | 0.15910824555422898 | No Hit |
| GTGTTCCAGACAGGACATGCTACTAAA | 10981 | 0.15801461919426502 | No Hit |
| GGTATCCAGACAGGACATGCTACTAAA | 10905 | 0.15692099283430108 | No Hit |
| ACCTCTTCCAGACAGGACATGCTACTA | 10782 | 0.1551510449096226 | No Hit |
| GTGGGCCAGACAGGACATGCTACTAAA | 10513 | 0.1512801831881712 | No Hit |
| GGTTCCAGACAGGACATGCTACTAAAT | 10504 | 0.15115067480343866 | No Hit |
| TCATTCCAGACAGGACATGCTACTAAA | 10487 | 0.15090604785449935 | No Hit |
| CCGACACTCTTTCCCAGACAGGACATG | 10292 | 0.14810003285196027 | No Hit |
| GGGTCACCAGACAGGACATGCTACTAA | 10278 | 0.14789857536459847 | No Hit |
| GGGCCGACCAGACAGGACATGCTACTA | 10172 | 0.1463732543888593 | No Hit |
| GGGGATCCAGACAGGACATGCTACTAA | 10081 | 0.1450637807210077 | No Hit |
| GGTGTCGGCCAGACAGGACATGCTACT | 10026 | 0.14427234059208643 | No Hit |
| GGGGTCGGCCAGACAGGACATGCTACT | 9882 | 0.14220020643636525 | No Hit |
| GGTGTACCAGACAGGACATGCTACTAA | 9771 | 0.14060293635799687 | No Hit |
| GGGACCAGACAGGACATGCTACTAAAT | 9715 | 0.13979710640854973 | No Hit |
| GGAAGAGCGTCGCCAGACAGGACATGC | 9712 | 0.13975393694697222 | No Hit |
| GTGGTATCCAGACAGGACATGCTACTA | 9643 | 0.13876103933068915 | No Hit |
| GTGGCGCCAGACAGGACATGCTACTAA | 9624 | 0.13848763274069817 | No Hit |
| GGTGCGTCCAGACAGGACATGCTACTA | 9541 | 0.13729327763705332 | No Hit |
| AGACAGGACATGCTACTAAATCGTATG | 9450 | 0.13598380396920176 | No Hit |
| CGGCCGAACACTCTTTCCCAGACAGGA | 9403 | 0.13530748240448723 | No Hit |
| GGTGGTCGGTTCACCAGACAGGACATG | 9260 | 0.13324973806929188 | No Hit |
| ACCGAACACTCTTTCCCAGACAGGACA | 9241 | 0.13297633147930088 | No Hit |
| GGCCGCCAGACAGGACATGCTACTAAA | 9197 | 0.13234317937616386 | No Hit |
| ACACTCTTCCCAGACAGGACATGCTAC | 9103 | 0.13099053624673476 | No Hit |
| GGTGGCGTACACCAGACAGGACATGCT | 9087 | 0.13076029911832132 | No Hit |
| GGTGGTCGGCCAGACAGGACATGCTAC | 9081 | 0.13067396019516628 | No Hit |
| GGTGGGCGTATCACCAGACAGGACATG | 9068 | 0.13048689252833032 | No Hit |
| CCACACCGAACACTCTTTCCAGACAGG | 8981 | 0.1292349781425821 | No Hit |
| TCACCGAACACTCTTTCCAGACAGGAC | 8731 | 0.12563752301112174 | No Hit |
| GGAAGCGAACCAGACAGGACATGCTAC | 8716 | 0.12542167570323412 | No Hit |
| GGTGGTCGCCGCCAGACAGGACATGCT | 8682 | 0.12493242180535552 | No Hit |
| GGTGCGGCCAGACAGGACATGCTACTA | 8532 | 0.1227739487264793 | No Hit |
| ACTCTTTCCCAGACAGGACATGCTACT | 8471 | 0.12189616967440296 | No Hit |
| GTGTCCAGACAGGACATGCTACTAAAT | 8402 | 0.12090327205811992 | No Hit |
| ATTCAGCTCATGTATCTTTGCTCATGC | 8377 | 0.12054352654497387 | No Hit |
| GGCGGCCAGACAGGACATGCTACTAAA | 8364 | 0.12035645887813794 | No Hit |
| GAAGACGTCGTAGAGCCAGACAGGACA | 8360 | 0.12029889959603457 | No Hit |
| GTGCACCAGACAGGACATGCTACTAAA | 8355 | 0.12022695049340534 | No Hit |
| GTGTCGTACACCAGACAGGACATGCTA | 8351 | 0.12016939121130199 | No Hit |
| GGTGGTCGGTACCAGACAGGACATGCT | 8235 | 0.1185001720303044 | No Hit |
| ACGGCACCACCGAACACTCTTTCCCAG | 8220 | 0.11828432472241676 | No Hit |
| GGTGGTCGCCCCAGACAGGACATGCTA | 8215 | 0.11821237561978756 | No Hit |
| ATTGTTGTTTTACCCAGACAGGACATG | 8192 | 0.11788140974769319 | No Hit |
| GGTGCGACCAGACAGGACATGCTACTA | 8189 | 0.11783824028611567 | No Hit |
| GGTGGGTCCAGACAGGACATGCTACTA | 8095 | 0.11648559715668658 | No Hit |
| CTGCAGCGGAGCAGAACGCTGTGAAAG | 8013 | 0.11530563187356759 | No Hit |
| GGGGGCACCAGACAGGACATGCTACTA | 7972 | 0.11471564923200808 | No Hit |
| GGTGGTCGGACCAGACAGGACATGCTA | 7891 | 0.11355007376941492 | No Hit |
| CATCTTCCCAGACAGGACATGCTACTA | 7876 | 0.11333422646152731 | No Hit |
| GGTGGACCCTCTACAATACCCTCTCCA | 7814 | 0.11244205758892513 | No Hit |
| CCAGACAGGACATGCTACTAAATCGTA | 7787 | 0.1120535324347274 | No Hit |
| GGTGGTCGTACCAGACAGGACATGCTA | 7700 | 0.1108016180489792 | No Hit |
| ACATGAGGGTAAATAATTTTGACTTCA | 7665 | 0.11029797433057476 | No Hit |
| GGTGGGCACCAGACAGGACATGCTACT | 7662 | 0.11025480486899722 | No Hit |
| GGTCGTATCCAGACAGGACATGCTACT | 7634 | 0.10985188989427368 | No Hit |
| GGTGTCGCCAGACAGGACATGCTACTA | 7626 | 0.10973677133006693 | No Hit |
| GGGGCGGCCAGACAGGACATGCTACTA | 7575 | 0.10900289048324903 | No Hit |
| CCCCGAACACTCTTTCCCAGACAGGAC | 7505 | 0.10799560304644011 | No Hit |
| AAACCAGACAGGACATGCTACTAAATC | 7486 | 0.10772219645644913 | No Hit |
| GGAGCCAGACAGGACATGCTACTAAAT | 7391 | 0.10635516350649421 | No Hit |
| GGGGGACCAGACAGGACATGCTACTAA | 7331 | 0.1054917742749437 | No Hit |
| GGTGGCGACCAGACAGGACATGCTACT | 7321 | 0.1053478760696853 | No Hit |
| GGGTTCCAGACAGGACATGCTACTAAA | 7317 | 0.10529031678758194 | No Hit |
| GAATATCCAGACAGGACATGCTACTAA | 7297 | 0.10500252037706509 | No Hit |
| GGAAGAGGGCCAGACAGGACATGCTAC | 7234 | 0.1040959616839371 | No Hit |
| AATGATACACTCTTTCCCAGACAGGAC | 7181 | 0.10333330119606748 | No Hit |
| CTCTTCCCAGACAGGACATGCTACTAA | 7136 | 0.10268575927240463 | No Hit |
| GTGGTCCGTTCACCAGACAGGACATGC | 7128 | 0.1025706407081979 | No Hit |
| CCCGAACATCTTTCCAGACAGGACATG | 7095 | 0.10209577663084513 | No Hit |
| GGTGTCACCAGACAGGACATGCTACTA | 7087 | 0.10198065806663839 | No Hit |
| GTGCGCCGCCAGACAGGACATGCTACT | 7075 | 0.1018079802203283 | No Hit |
| GGGTGATCCAGACAGGACATGCTACTA | 7067 | 0.10169286165612158 | No Hit |
| GTGGCTCCAGACAGGACATGCTACTAA | 7062 | 0.10162091255349236 | No Hit |
| GGTGGCCCAGACAGGACATGCTACTAA | 7023 | 0.10105970955298453 | No Hit |
| GGCACCAGACAGGACATGCTACTAAAT | 7016 | 0.10095898080930364 | No Hit |
| GGGCGACCAGACAGGACATGCTACTAA | 6986 | 0.1005272861935284 | No Hit |
| GGGATCCAGACAGGACATGCTACTAAA | 6973 | 0.10034021852669246 | No Hit |
| GGGCACCAGACAGGACATGCTACTAAA | 6971 | 0.10031143888564079 | No Hit |
| GTGACCAGACAGGACATGCTACTAAAT | 6957 | 0.10010998139827901 | No Hit |

## Adapter Content

## Kmer Content

| Sequence | Count | PValue | Obs/Exp Max | Max Obs/Exp Position |
| --- | --- | --- | --- | --- |
| TTTCGGG | 25 | 0.0033791238 | 21.002535 | 21 |
| ATCAGGG | 40 | 5.4112497E-6 | 21.002535 | 21 |
| TAAACGC | 85 | 0.0 | 21.002535 | 21 |
| ACCTGAA | 25 | 0.0033791238 | 21.002535 | 21 |
| TCGTCTG | 25 | 0.0033791238 | 21.002535 | 21 |
| ACTGTAT | 25 | 0.0033791238 | 21.002535 | 21 |
| GCAGCAT | 25 | 0.0033791238 | 21.002535 | 21 |
| TATACGA | 25 | 0.0033791238 | 21.002535 | 21 |
| ATCGTAG | 30 | 3.9331822E-4 | 21.002533 | 21 |
| CTCTCAA | 30 | 3.9331822E-4 | 21.002533 | 21 |
| GGTTAGT | 45 | 6.3874904E-7 | 21.002533 | 21 |
| ATCGCAT | 90 | 0.0 | 21.002533 | 21 |
| TCAACGA | 60 | 1.0713848E-9 | 21.002533 | 21 |
| TCAAAGC | 75 | 1.8189894E-12 | 21.002533 | 21 |
| CCCCTGT | 190 | 0.0 | 21.00208 | 18 |
| ATCTATA | 55 | 8.991265E-9 | 21.00208 | 15 |
| TAAAGGC | 310 | 0.0 | 21.00208 | 15 |
| CTTCATT | 25 | 0.0033795468 | 21.00208 | 15 |
| CACATTT | 55 | 8.991265E-9 | 21.00208 | 18 |
| GAGTATT | 25 | 0.0033795468 | 21.00208 | 15 |

Produced by FastQC (version 0.11.5)
