# Supplementary material for: Multidimensional chromatin profiling of zebrafish pancreas to uncover and investigate disease-relevant enhancers
Source: Nat Commun. 2022 Apr 11;13:1945. doi: 10.1038/s41467-022-29551-7 (PMC9001708; doi:10.1038/s41467-022-29551-7)
Supplement: Supplementary file 3 — Supplementary data1-17 [file 41467_2022_29551_MOESM3_ESM.zip › SupplementaryFile1_FASTQC_reports/Supplementary data 6_Pancreas H3K4me3 HiChIP fastqc 1-1 .html]

FCHHWFYBBXX\_L3\_CHKPEI85217070034\_1.fq FastQC Report 

FastQC Report

Wed 15 Apr 2020  
FCHHWFYBBXX\_L3\_CHKPEI85217070034\_1.fq

## Summary

- Basic Statistics
- Per base sequence quality
- Per tile sequence quality
- Per sequence quality scores
- Per base sequence content
- Per sequence GC content
- Per base N content
- Sequence Length Distribution
- Sequence Duplication Levels
- Overrepresented sequences
- Adapter Content
- Kmer Content

## Basic Statistics

| Measure | Value |
| --- | --- |
| Filename | FCHHWFYBBXX\_L3\_CHKPEI85217070034\_1.fq |
| File type | Conventional base calls |
| Encoding | Sanger / Illumina 1.9 |
| Total Sequences | 100736229 |
| Sequences flagged as poor quality | 0 |
| Sequence length | 49 |
| %GC | 46 |

## Per base sequence quality

## Per tile sequence quality

## Per sequence quality scores

## Per base sequence content

## Per sequence GC content

## Per base N content

## Sequence Length Distribution

## Sequence Duplication Levels

## Overrepresented sequences

| Sequence | Count | Percentage | Possible Source |
| --- | --- | --- | --- |
| GTGTGTGTGTGTGTGTGTGTGTGTGTGTGTGTGTGTGTGTGTGTGTGTG | 829301 | 0.8232400678806431 | No Hit |
| CACACACACACACACACACACACACACACACACACACACACACACACAC | 658175 | 0.6533647393134003 | No Hit |
| CACACACACACACACACACACCTGTCTCTTATACACATCTCCGAGCCCA | 184300 | 0.18295304661444098 | No Hit |
| CACACACACACACACACACACACCTGTCTCTTATACACATCTCCGAGCC | 180006 | 0.17869042923971276 | No Hit |
| GTGTGTGTGTGTGTGTGTGTGTGCTGTCTCTTATACACATCTCCGAGCC | 166783 | 0.16556406930817313 | No Hit |
| GTGTGTGTGTGTGTGTGTGTGCTGTCTCTTATACACATCTCCGAGCCCA | 161336 | 0.16015687861414785 | No Hit |
| CACACACACACACACACACACACACCTGTCTCTTATACACATCTCCGAG | 157039 | 0.15589128316486814 | No Hit |
| GTGTGTGTGTGTGTGTGTGTGTGTGCTGTCTCTTATACACATCTCCGAG | 151125 | 0.1500205055323244 | No Hit |
| CCCTAACCCTAACCCTAACCCTAACCCTAACCCTAACCCTAACCCTAAC | 150573 | 0.14947253981484654 | No Hit |
| GGGTTAGGGTTAGGGTTAGGGTTAGGGTTAGGGTTAGGGTTAGGGTTAG | 150153 | 0.14905560937763512 | No Hit |
| CACACACACACACACACACCTGTCTCTTATACACATCTCCGAGCCCACG | 135683 | 0.13469136312418445 | No Hit |
| GTGTGTGTGTGTGTGTGTGTGTGTGTGCTGTCTCTTATACACATCTCCG | 133905 | 0.1329263576066561 | No Hit |
| CACACACACACACACACACACACACACCTGTCTCTTATACACATCTCCG | 133838 | 0.13285984727500572 | No Hit |
| GTGTGTGTGTGTGTGTGTGCTGTCTCTTATACACATCTCCGAGCCCACG | 116635 | 0.11578257510512927 | No Hit |
| GTGTGTGTGTGTGTGTGTGTGTGTGTGTGCTGTCTCTTATACACATCTC | 115444 | 0.11460027950817972 | No Hit |
| CACACACACACACACACACACACACACACCTGTCTCTTATACACATCTC | 113396 | 0.11256724728101546 | No Hit |
| GTGTGTGTGTGTGTGTGTGTGTGTGTGTGTGCTGTCTCTTATACACATC | 101370 | 0.10062913909552838 | No Hit |

## Adapter Content

## Kmer Content

| Sequence | Count | PValue | Obs/Exp Max | Max Obs/Exp Position |
| --- | --- | --- | --- | --- |
| GCCCACG | 48080 | 0.0 | 24.739302 | 43 |
| AGCCCAC | 54065 | 0.0 | 22.127808 | 42 |
| CCCACGA | 20320 | 0.0 | 19.663761 | 42 |
| CCACGAG | 20495 | 0.0 | 19.495861 | 43 |
| CGAGCCC | 101690 | 0.0 | 17.60356 | 42 |
| GAGCCCA | 102125 | 0.0 | 17.46337 | 43 |
| CCGAGCC | 139605 | 0.0 | 12.991916 | 43 |
| GGGTATA | 7735 | 0.0 | 12.989829 | 1 |
| TCCGAGC | 144055 | 0.0 | 12.641285 | 42 |
| CTATACT | 35255 | 0.0 | 12.290533 | 4 |
| CCTATAC | 30905 | 0.0 | 11.865576 | 3 |
| TATACTG | 45755 | 0.0 | 11.63913 | 5 |
| GTCCTAC | 8755 | 0.0 | 11.476452 | 1 |
| TAACAGT | 49970 | 0.0 | 11.448468 | 4 |
| GTATTAA | 20805 | 0.0 | 11.306674 | 1 |
| TTATACT | 19090 | 0.0 | 11.298306 | 4 |
| GTACTAA | 14700 | 0.0 | 11.162081 | 1 |
| GCCTATA | 29860 | 0.0 | 11.038996 | 2 |
| GGCCTAT | 32050 | 0.0 | 10.911907 | 1 |
| GTATTAT | 22160 | 0.0 | 10.90721 | 1 |

Produced by FastQC (version 0.11.5)
