# Supplementary material for: Multidimensional chromatin profiling of zebrafish pancreas to uncover and investigate disease-relevant enhancers
Source: Nat Commun. 2022 Apr 11;13:1945. doi: 10.1038/s41467-022-29551-7 (PMC9001708; doi:10.1038/s41467-022-29551-7)
Supplement: Supplementary file 3 — Supplementary data1-17 [file 41467_2022_29551_MOESM3_ESM.zip › SupplementaryFile1_FASTQC_reports/Supplementary data 7_Pancreas H3K4me3 HiChIP fastqc 1-2 .html]

FCHHWFYBBXX\_L3\_CHKPEI85217070034\_2.fq FastQC Report 

FastQC Report

Wed 15 Apr 2020  
FCHHWFYBBXX\_L3\_CHKPEI85217070034\_2.fq

## Summary

- Basic Statistics
- Per base sequence quality
- Per tile sequence quality
- Per sequence quality scores
- Per base sequence content
- Per sequence GC content
- Per base N content
- Sequence Length Distribution
- Sequence Duplication Levels
- Overrepresented sequences
- Adapter Content
- Kmer Content

## Basic Statistics

| Measure | Value |
| --- | --- |
| Filename | FCHHWFYBBXX\_L3\_CHKPEI85217070034\_2.fq |
| File type | Conventional base calls |
| Encoding | Sanger / Illumina 1.9 |
| Total Sequences | 100736229 |
| Sequences flagged as poor quality | 0 |
| Sequence length | 49 |
| %GC | 46 |

## Per base sequence quality

## Per tile sequence quality

## Per sequence quality scores

## Per base sequence content

## Per sequence GC content

## Per base N content

## Sequence Length Distribution

## Sequence Duplication Levels

## Overrepresented sequences

| Sequence | Count | Percentage | Possible Source |
| --- | --- | --- | --- |
| GTGTGTGTGTGTGTGTGTGTGTGTGTGTGTGTGTGTGTGTGTGTGTGTG | 794435 | 0.7886288854429919 | No Hit |
| CACACACACACACACACACACACACACACACACACACACACACACACAC | 625223 | 0.6206535684396127 | No Hit |
| GTGTGTGTGTGTGTGTGTGTGCTGTCTCTTATACACATCTGACGCTGCC | 186518 | 0.18515483639952415 | No Hit |
| GTGTGTGTGTGTGTGTGTGTGTGCTGTCTCTTATACACATCTGACGCTG | 183441 | 0.18210032460119188 | No Hit |
| CACACACACACACACACACACACCTGTCTCTTATACACATCTGACGCTG | 166962 | 0.16574176108974656 | No Hit |
| CACACACACACACACACACACCTGTCTCTTATACACATCTGACGCTGCC | 163188 | 0.1619953433039468 | No Hit |
| GTGTGTGTGTGTGTGTGTGTGTGTGCTGTCTCTTATACACATCTGACGC | 160547 | 0.1593736450071007 | No Hit |
| CACACACACACACACACACACACACCTGTCTCTTATACACATCTGACGC | 151611 | 0.15050295360966906 | No Hit |
| GGGTTAGGGTTAGGGTTAGGGTTAGGGTTAGGGTTAGGGTTAGGGTTAG | 137753 | 0.13674623456472645 | No Hit |
| GTGTGTGTGTGTGTGTGTGCTGTCTCTTATACACATCTGACGCTGCCGA | 136710 | 0.1357108573123181 | No Hit |
| GTGTGTGTGTGTGTGTGTGTGTGTGTGCTGTCTCTTATACACATCTGAC | 135839 | 0.13484622300086296 | No Hit |
| CCCTAACCCTAACCCTAACCCTAACCCTAACCCTAACCCTAACCCTAAC | 133431 | 0.13245582182751747 | No Hit |
| CACACACACACACACACACACACACACCTGTCTCTTATACACATCTGAC | 132184 | 0.13121793550560643 | No Hit |
| CACACACACACACACACACCTGTCTCTTATACACATCTGACGCTGCCGA | 117816 | 0.11695494378690709 | No Hit |
| GTGTGTGTGTGTGTGTGTGTGTGTGTGTGCTGTCTCTTATACACATCTG | 116314 | 0.11546392112811767 | No Hit |
| CACACACACACACACACACACACACACACCTGTCTCTTATACACATCTG | 111409 | 0.11059476923639855 | No Hit |

## Adapter Content

## Kmer Content

| Sequence | Count | PValue | Obs/Exp Max | Max Obs/Exp Position |
| --- | --- | --- | --- | --- |
| CTGCCGA | 54185 | 0.0 | 22.284218 | 43 |
| GCCGACG | 20980 | 0.0 | 19.1913 | 43 |
| TGCCGAC | 21375 | 0.0 | 18.936535 | 42 |
| GCTGCCG | 67410 | 0.0 | 18.11896 | 42 |
| ACGCTGC | 101325 | 0.0 | 17.682568 | 42 |
| CGCTGCC | 104150 | 0.0 | 17.341852 | 43 |
| GGGTATA | 7520 | 0.0 | 12.68252 | 1 |
| TGACGCT | 146895 | 0.0 | 12.526307 | 42 |
| GACGCTG | 148615 | 0.0 | 12.339835 | 43 |
| CTATACT | 35360 | 0.0 | 11.767328 | 4 |
| TAACAGT | 49410 | 0.0 | 11.466088 | 4 |
| GTACTAA | 14195 | 0.0 | 11.427998 | 1 |
| TATACTG | 44500 | 0.0 | 11.191405 | 5 |
| GTATTAT | 21690 | 0.0 | 11.133233 | 1 |
| GTCCTAC | 8780 | 0.0 | 10.986479 | 1 |
| CCTATAC | 30655 | 0.0 | 10.930203 | 3 |
| GTATTAA | 20525 | 0.0 | 10.789146 | 1 |
| GTATAAA | 29070 | 0.0 | 10.568935 | 1 |
| TTATACT | 18940 | 0.0 | 10.553287 | 4 |
| GGCCTAT | 32320 | 0.0 | 10.483047 | 1 |

Produced by FastQC (version 0.11.5)
