# Supplementary material for: Multidimensional chromatin profiling of zebrafish pancreas to uncover and investigate disease-relevant enhancers
Source: Nat Commun. 2022 Apr 11;13:1945. doi: 10.1038/s41467-022-29551-7 (PMC9001708; doi:10.1038/s41467-022-29551-7)
Supplement: Supplementary file 3 — Supplementary data1-17 [file 41467_2022_29551_MOESM3_ESM.zip › SupplementaryFile1_FASTQC_reports/Supplementary data 8_ Pancreas H3K4me3 HiChIP fastqc 2-1 .html]

FCHHWFYBBXX\_L3\_CHKPEI85217070035\_1.fq FastQC Report 

FastQC Report

Wed 15 Apr 2020  
FCHHWFYBBXX\_L3\_CHKPEI85217070035\_1.fq

## Summary

- Basic Statistics
- Per base sequence quality
- Per tile sequence quality
- Per sequence quality scores
- Per base sequence content
- Per sequence GC content
- Per base N content
- Sequence Length Distribution
- Sequence Duplication Levels
- Overrepresented sequences
- Adapter Content
- Kmer Content

## Basic Statistics

| Measure | Value |
| --- | --- |
| Filename | FCHHWFYBBXX\_L3\_CHKPEI85217070035\_1.fq |
| File type | Conventional base calls |
| Encoding | Sanger / Illumina 1.9 |
| Total Sequences | 81119211 |
| Sequences flagged as poor quality | 0 |
| Sequence length | 49 |
| %GC | 46 |

## Per base sequence quality

## Per tile sequence quality

## Per sequence quality scores

## Per base sequence content

## Per sequence GC content

## Per base N content

## Sequence Length Distribution

## Sequence Duplication Levels

## Overrepresented sequences

| Sequence | Count | Percentage | Possible Source |
| --- | --- | --- | --- |
| GTGTGTGTGTGTGTGTGTGTGTGTGTGTGTGTGTGTGTGTGTGTGTGTG | 577823 | 0.7123133877621172 | No Hit |
| CACACACACACACACACACACACACACACACACACACACACACACACAC | 401416 | 0.49484702211908843 | No Hit |

## Adapter Content

## Kmer Content

| Sequence | Count | PValue | Obs/Exp Max | Max Obs/Exp Position |
| --- | --- | --- | --- | --- |
| GCCCACG | 14955 | 0.0 | 15.513817 | 43 |
| CGAGCCC | 27950 | 0.0 | 12.835592 | 42 |
| CCGAGCC | 34360 | 0.0 | 12.229171 | 43 |
| TATACTG | 72610 | 0.0 | 11.674778 | 5 |
| TCCGAGC | 36620 | 0.0 | 11.574175 | 42 |
| CTGTTAA | 54830 | 0.0 | 11.570111 | 1 |
| GTATTAT | 22970 | 0.0 | 11.542759 | 1 |
| TCTATAC | 13875 | 0.0 | 11.441899 | 3 |
| CTATACT | 65150 | 0.0 | 11.422245 | 4 |
| TAACAGT | 79400 | 0.0 | 11.420441 | 4 |
| GTATAGG | 47430 | 0.0 | 11.293765 | 1 |
| GTATTAG | 15025 | 0.0 | 11.219094 | 1 |
| GTATTAA | 22350 | 0.0 | 11.178187 | 1 |
| GTGTAGG | 24355 | 0.0 | 10.877503 | 1 |
| CCTATAC | 57790 | 0.0 | 10.843523 | 3 |
| GTATAGA | 14875 | 0.0 | 10.839521 | 1 |
| GAGCCCA | 33435 | 0.0 | 10.826296 | 43 |
| TATAGGC | 43595 | 0.0 | 10.75981 | 2 |
| ATAGGCC | 44935 | 0.0 | 10.752051 | 3 |
| TAATACT | 18785 | 0.0 | 10.738469 | 4 |

Produced by FastQC (version 0.11.5)
