# Supplementary material for: Multidimensional chromatin profiling of zebrafish pancreas to uncover and investigate disease-relevant enhancers
Source: Nat Commun. 2022 Apr 11;13:1945. doi: 10.1038/s41467-022-29551-7 (PMC9001708; doi:10.1038/s41467-022-29551-7)
Supplement: Supplementary file 3 — Supplementary data1-17 [file 41467_2022_29551_MOESM3_ESM.zip › SupplementaryFile1_FASTQC_reports/Supplementary data 9_Pancreas H3K4me3 HiChIP fastqc 2-2 .html]

FCHHWFYBBXX\_L3\_CHKPEI85217070035\_2.fq FastQC Report 

FastQC Report

Thu 16 Apr 2020  
FCHHWFYBBXX\_L3\_CHKPEI85217070035\_2.fq

## Summary

- Basic Statistics
- Per base sequence quality
- Per tile sequence quality
- Per sequence quality scores
- Per base sequence content
- Per sequence GC content
- Per base N content
- Sequence Length Distribution
- Sequence Duplication Levels
- Overrepresented sequences
- Adapter Content
- Kmer Content

## Basic Statistics

| Measure | Value |
| --- | --- |
| Filename | FCHHWFYBBXX\_L3\_CHKPEI85217070035\_2.fq |
| File type | Conventional base calls |
| Encoding | Sanger / Illumina 1.9 |
| Total Sequences | 81119211 |
| Sequences flagged as poor quality | 0 |
| Sequence length | 49 |
| %GC | 46 |

## Per base sequence quality

## Per tile sequence quality

## Per sequence quality scores

## Per base sequence content

## Per sequence GC content

## Per base N content

## Sequence Length Distribution

## Sequence Duplication Levels

## Overrepresented sequences

| Sequence | Count | Percentage | Possible Source |
| --- | --- | --- | --- |
| GTGTGTGTGTGTGTGTGTGTGTGTGTGTGTGTGTGTGTGTGTGTGTGTG | 585710 | 0.7220361154646833 | No Hit |
| CACACACACACACACACACACACACACACACACACACACACACACACAC | 385430 | 0.47514022294916053 | No Hit |

## Adapter Content

## Kmer Content

| Sequence | Count | PValue | Obs/Exp Max | Max Obs/Exp Position |
| --- | --- | --- | --- | --- |
| CTGCCGA | 17895 | 0.0 | 13.610623 | 43 |
| TATACTG | 71875 | 0.0 | 12.069704 | 5 |
| CTATACT | 64115 | 0.0 | 11.980698 | 4 |
| GTATTAT | 23150 | 0.0 | 11.723522 | 1 |
| TCTATAC | 14050 | 0.0 | 11.549307 | 3 |
| CTGTTAA | 54360 | 0.0 | 11.48666 | 1 |
| ACGCTGC | 31955 | 0.0 | 11.483061 | 42 |
| TAACAGT | 79875 | 0.0 | 11.425003 | 4 |
| GTATTAG | 14635 | 0.0 | 11.391442 | 1 |
| CCTATAC | 57390 | 0.0 | 11.291103 | 3 |
| GTATAGA | 14850 | 0.0 | 11.197204 | 1 |
| TGACGCT | 39230 | 0.0 | 11.101562 | 42 |
| CGCTGCC | 33795 | 0.0 | 10.966414 | 43 |
| GTATTAA | 21925 | 0.0 | 10.95903 | 1 |
| GTATAGG | 48290 | 0.0 | 10.94745 | 1 |
| GTCATAG | 20220 | 0.0 | 10.882098 | 1 |
| ATACTGC | 64205 | 0.0 | 10.780017 | 6 |
| GTGTAGG | 23830 | 0.0 | 10.731401 | 1 |
| GTTAAAC | 57425 | 0.0 | 10.715333 | 3 |
| TTATACT | 21005 | 0.0 | 10.661842 | 4 |

Produced by FastQC (version 0.11.5)
