# Supplementary material for: Multidimensional chromatin profiling of zebrafish pancreas to uncover and investigate disease-relevant enhancers
Source: Nat Commun. 2022 Apr 11;13:1945. doi: 10.1038/s41467-022-29551-7 (PMC9001708; doi:10.1038/s41467-022-29551-7)
Supplement: Supplementary file 9 — Reporting Summary [file 41467_2022_29551_MOESM9_ESM.pdf]

nature research

Corresponding author(s): José Bessa
Last updated by author(s): Jan 10, 2022

Reporting Summary

Nature Research wishes to improve the reproducibility of the work that we publish. This form provides structure for consistency and transparency in reporting. For further information on Nature Research policies, see our Editorial Policies and the Editorial Policy Checklist.

Statistics

For all statistical analyses, confirm that the following items are present in the figure legend, table legend, main text, or Methods section.

n/a	Confirmed	
☐	☒	The exact sample size (*n*) for each experimental group/condition, given as a discrete number and unit of measurement
☐	☒	A statement on whether measurements were taken from distinct samples or whether the same sample was measured repeatedly
☐	☒	The statistical test(s) used AND whether they are one- or two-sided<br>*Only common tests should be described solely by name; describe more complex techniques in the Methods section.*
☒	☐	A description of all covariates tested
☐	☒	A description of any assumptions or corrections, such as tests of normality and adjustment for multiple comparisons
☐	☒	A full description of the statistical parameters including central tendency (e.g. means) or other basic estimates (e.g. regression coefficient) AND variation (e.g. standard deviation) or associated estimates of uncertainty (e.g. confidence intervals)
☐	☒	For null hypothesis testing, the test statistic (e.g. *F*, *t*, *r*) with confidence intervals, effect sizes, degrees of freedom and *P* value noted<br>*Give P values as exact values whenever suitable.*
☒	☐	For Bayesian analysis, information on the choice of priors and Markov chain Monte Carlo settings
☒	☐	For hierarchical and complex designs, identification of the appropriate level for tests and full reporting of outcomes
☐	☒	Estimates of effect sizes (e.g. Cohen's *d*, Pearson's *r*), indicating how they were calculated

Our web collection on statistics for biologists contains articles on many of the points above.

Software and code

Policy information about availability of computer code

Data collection	List of softwares:<br>-LAS AF software (v.2.6.3.8173-Leica-SP5II confocal microscope)<br>-LAS AF software (v.3.7.4.23463-LeicaDMI6000 FFW microscope)<br>-LAS AF software (v.2.0.0.14332-Leica m205 stereomicroscope)
Data analysis	List of softwares:<br>Images processing:<br>-ImageJ Java (1.8.0)<br>-GraphPad Prism 7 (v.7.04)<br>-Adobe Illustrator CC 2018<br><br>Genome-wide analysis:<br>-FASTQC (v.0.11.5)<br>-SKEWER (v.0.2.1)<br>-Bowtie (v1.1.2)<br>-Bowtie2 (v.2.2.6)<br>-BWA-MEM (v.0.7.17)<br>-MACS14 (v.14.2)<br>-HOMER (v.4.11.1)<br>-seqMINER (v1.3.4)<br>-Bedtools (v.2.27)<br>-samtools (v.1.9)<br>-MACS2 (v.2.1.0)<br>-Bowtie (v1.1.2)

-IDR (v.2.0.4)
-HT-seq-count(v.0.9.0)
-HOMER(v.4.8)
-ROSE (V.1)
-seqMINER (v1.3.4)
-GREAT (v.3.0.0)
-PANTHER (v.14.0)
-bedGraphToBigWig (v.1.04.00)
-genomeCoverageBed (v.1.04.00)
-liftOver (v.1.04.00)
-samtools (v.1.9)
-bedClip (v.1.04.00)
-DisGeNET (v.6.0)
-GEM-Mapper(v.3.6)

For manuscripts utilizing custom algorithms or software that are central to the research but not yet described in published literature, software must be made available to editors and reviewers. We strongly encourage code deposition in a community repository (e.g. GitHub). See the Nature Research guidelines for submitting code & software for further information.

Data

Policy information about availability of data

All manuscripts must include a data availability statement. This statement should provide the following information, where applicable:

- Accession codes, unique identifiers, or web links for publicly available datasets
- A list of figures that have associated raw data
- A description of any restrictions on data availability

All sequencing data generated within this study has been submitted to European Nucleotide Archive (ENA) browser under accession number PRJEB40292 (https://www.ebi.ac.uk/ena/browser/view/PRJEB40292). The analysed data are available on USCS browser (http://genome-euro.ucsc.edu/s/VDR_group_public_data/Carrico_et_al_2020_ZebrafishPancreasRegulome).
Other datasets used in this study can be downloaded from ENCODE project (https://www.encodeproject.org/): ChIP-seq and ATAC-seq of Human pancreas (https://www.encodeproject.org/ENCSR340GAZ), ChIP-seq and ATAC-seq of left ventricle(https://www.encodeproject.org/ENCSR464TTP); from Expression Atlas: data(http://www.ebi.ac.uk/gxa/experiments/): RNA-seq of zebrafish development stages (http://www.ebi.ac.uk/gxa/experiments/E-ERAD-475); NCBI Gene Expression Omnibus (GEO)(https://www.ncbi.nlm.nih.gov/geo/): ChIp-seq of developmental stages of zebrafish (https://www.ncbi.nlm.nih.gov/geo/GSE32483); European Nucleotide Archive (ENA) browser: RNAseq of the pancreatic acinar, alpha, beta and delta cells from zebrafish (https://www.ebi.ac.uk/ena/browser/view/PRJEB10140), RNA-seq of developmental stages of zebrafish (https://www.ebi.ac.uk/ena/browser/view/PRJEB12296; https://www.ebi.ac.uk/ena/browser/view/PRJEB7244; https://www.ebi.ac.uk/ena/browser/view/PRJEB12982).
All other relevant data supporting the key findings of this study are available within the article and its Supplementary Information files(supplementary Fig1-10; supplementary table1-4 supplementary data1-17) or from the corresponding author upon reasonable request.
No data availability restrictions.
The custom code for analysis is available under https://gitlab.com/rdacemel/pancreasregulome
Figures that have associated raw data: Figure1, Figure2, Figure3, Figure4, Figure5, FigureS1, FigureS2, FigureS3, FigureS4, FigureS5, FigureS6, FigureS7, FigureS8, FigureS9 and FigureS10.

Field-specific reporting

Please select the one below that is the best fit for your research. If you are not sure, read the appropriate sections before making your selection.

☒ Life sciences ☐ Behavioural & social sciences ☐ Ecological, evolutionary & environmental sciences

For a reference copy of the document with all sections, see nature.com/documents/nr-reporting-summary-flat.pdf

Life sciences study design

All studies must disclose on these points even when the disclosure is negative.

Sample size	No statistical methodology was calculated in this study to determine the necessary sample size for the following experiments. However, we decided on replicate number based on previous publications which had success seeing statistical significance for measure of effective similar to what we wished to what we achieve with these experiments. These replicate numbers were based on the relative variance in the assay as well as the effective size observed.<br><br>Experiments using zebrafish embryos: The number of animals used for sample preparation was determined following previously reported methods, and allowed the extraction of enough biological material to perform the experiment(Eufrasio, E. el al 2020; PMID: 32912862; Amorim, J. el al 2020; PMID: 32640228);<br><br>Chromatin assays: For each technique was performed two biological replicates and the reproducibility was measured by Pearson's correlation coefficient(Yang, Y. et al 2014; PMID: 24688750, Bailey, T. el al 2008; PMID: 24244136).<br><br>Cell line experiments: For each tecnique using cell lines, three tecniqual replicates were performed (Pasquali, et al 2014; PMID: 24413736).
Data exclusions	No data were excluded.
Replication	For genome-wide studies data were collected in duplicates (H3K27ac ChIP-seq, H3K4me3 HiChIP-seq, ATAC-seq, 4C-seq and RNA-seq) from

Replication	pools of several fishes (n>=10); for the remaining assays, at least three were used. The reproducibility between the replicates was measured by Pearson's correlation coefficient. Good Pearson's correlations was obtained, so all attempts at replication were successful.
Randomization	Randomization of the samples is not applicable to our study, since no treatment conditions were compared. All comparisons were performed between different genotypes, which do not require randomization.
Blinding	Blinding was not relevant for our study, since comparisons were performed automatically using statistical software, not influenced by the researcher.

Reporting for specific materials, systems and methods

We require information from authors about some types of materials, experimental systems and methods used in many studies. Here, indicate whether each material, system or method listed is relevant to your study. If you are not sure if a list item applies to your research, read the appropriate section before selecting a response.

Materials & experimental systems

n/a	Involved in the study	
☐	☒	Antibodies
☐	☒	Eukaryotic cell lines
☒	☐	Palaeontology and archaeology
☐	☒	Animals and other organisms
☒	☐	Human research participants
☒	☐	Clinical data
☒	☐	Dual use research of concern

Methods

n/a	Involved in the study	
☐	☒	ChIP-seq
☐	☒	Flow cytometry
☒	☐	MRI-based neuroimaging

Antibodies

Antibodies used

List of antibodies used in this study:
-rabbit anti-H3K27ac antibody (1:2, #ab4729, Abcam)
-rabbit anti-H3K4me3 antibody (1:5, #ab8580, Abcam)
- rabbit anti-Amylase (1:50#A8273-1VL, Sigma-Aldrich)
- mouse anti-Alcam (1:50, #ZN-8, DSHB)
- mouse anti-Nkx6.1 (1:75, #F55A10, DSHB)
- goat anti-mouse AlexaFluor647 (1:800, #A-21236 Invitrogen, ThermoFisher Scientific)
- goat anti-rabbit AlexaFluor568 (1:800, #A-11036 Invitrogen, ThermoFisher Scientific)
- anti-ARID1A (1:1000 , #HPA005456 Sigma-Aldrich)
- donkey anti-rabbit (1:100, #A31573, ThermoFisher Scientific)
-DAPI (1:1000, D1306, Invitrogen, ThermoFisher Scientific)

Validation

The validation (species reactivity) of each primary antibody was described in the following websites and publications:
-rabbit anti-H3K27ac(https://www.abcam.com/histone-h3-acetyl-k27-antibody-chip-grade-ab4729.html; Shi, H. 2020; PMID: 32832616)
-rabbit anti-H3K4me3(https://www.abcam.com/histone-h3-tri-methyl-k4-antibody-chip-grade-ab8580.html; Lindeman, L. et al 2011; PMID: 22137762; Redl, S. 2021; PMID: 33298460).
- rabbit anti-Amylase (Park, S. et al 2008; PMID: 18549880)
-mouse anti-Alcam (https://dshb.biology.uiowa.edu/ZN-8; Otten, C. el al 2012; PMID: 22355335)
-mouse anti-Nkx6.1 (https://dshb.biology.uiowa.edu/F55A10; Kuscha, V. et al 2012; PMID: 22473852)
-anti-ARID1A (https://www.sigmaaldrich.com/catalog/product/sigma/hpa005456?lang=pt&region=PT)

Eukaryotic cell lines

Policy information about cell lines

Cell line source(s)	In this study was used the hTERT-HPNE human cell line (intermediary cells formed during acinar-to-ductal metaplasia; ATCC CRL-4023).
Authentication	The cells used were not authenticated.
Mycoplasma contamination	The hTERT-HPNE cell line, a human pancreatic duct cell line, was tested for mycoplasma contamination and the result was negative.
Commonly misidentified lines (See ICLAC register)	The study did not involve misidentified lines.

Animals and other organisms

Policy information about studies involving animals; ARRIVE guidelines recommended for reporting animal research

Laboratory animals	This study involved Adult zebrafish AB/TU WT strains, male and females, with 12-24 months.
Wild animals	The study did not involve wild animals.
Field-collected samples	The study did not involve field-collected samples.
Ethics oversight	The i3S animal facility and this project were licensed by Direcção Geral de Alimentação e Veterinária (DGAV) and all the protocols used for the experiments were approved by the i3S Animal Welfare and Ethics Review Body.

Note that full information on the approval of the study protocol must also be provided in the manuscript.

ChIP-seq

Data deposition

☒ Confirm that both raw and final processed data have been deposited in a public database such as GEO.

☒ Confirm that you have deposited or provided access to graph files (e.g. BED files) for the called peaks.

Data access links
May remain private before publication.

https://www.ebi.ac.uk/ena/browser/view/PRJEB40292

Files in database submission

ChIP-seq
Raw files:
Zebrafish_Whole_Pancreas_H3K27ac_ChIP-seq_replicate_1.fq Zebrafish_Whole_Pancreas_H3K27ac_ChIP-seq_replicate_2.fq
Processed signal files:
Whole_Pancreas_H3K27ac_ChIP-seq_replicate_1_danrer10.bw Whole_Pancreas_H3K27ac_ChIP-seq_replicate_2_danrer10.bw

ATAC-seq
Raw files:
Zebrafish_Whole_Pancreas_ATAC-seq_replicate_1_1.fq Zebrafish_Whole_Pancreas_ATAC-seq_replicate_1_2.fq
Zebrafish_Whole_Pancreas_ATAC-seq_replicate_2_1.fq Zebrafish_Whole_Pancreas_ATAC-seq_replicate_2_2.fq
Processed signal files:
Whole_Pancreas_ATAC-seq_replicate_1_danrer10.bw Whole_Pancreas_ATAC-seq_replicate_2_danrer10.bw

HiChIP-seq
Raw files:
Zebrafish_Whole_Pancreas_H3K4me3_HiChIP-seq_replicate_1_1.fq Zebrafish_Whole_Pancreas_H3K4me3_HiChIP-seq_replicate_1_2.fq
Zebrafish_Whole_Pancreas_H3K4me3_HiChIP-seq_replicate_2_1.fq Zebrafish_Whole_Pancreas_H3K4me3_HiChIP-seq_replicate_2_2.fq

Processed file: Whole_Pancreas_H3K4me3_HiChIP_landscapes_danrer10.bed

4C-se files
Raw files:
Zebrafish_Whole_Pancreas_4C-seq_Arid1ab_replicate_1_danrer10.fq Zebrafish_Whole_Pancreas_4C-seq_Arid1ab_replicate_2_danrer10.fq
Zebrafish_Whole_Pancreas_4C-seq_Ptf1a_replicate_1_danrer10.fq
Processed bedGraph files:
Zebrafish_Whole_Pancreas_4C-seq_Arid1ab_replicate1_danrer10.bedGraph Zebrafish_Whole_Pancreas_4C-seq_Arid1ab_replicate2_danrer10.bedGraph
Zebrafish_Whole_Pancreas_4C-seq_Ptf1a_replicate1_danrer10.bedGraph

RNA-seq
Raw files:
Zebrafish_Whole_Pancreas_RNA-seq_replicate_1.fq Zebrafish_Whole_Pancreas_RNA-seq_replicate_2.fq
Zebrafish_Endocrine_Pancreas_RNA-seq_replicate_1.fq
Zebrafish_Endocrine_Pancreas_RNA-seq_replicate_2.fq
Zebrafish_Endocrine_Pancreas_RNA-seq_replicate_3.fq
Zebrafish_Endocrine_Pancreas_RNA-seq_replicate_4.fq
Zebrafish_Muscle_RNA-seq_replicate_1.fq
Zebrafish_Muscle_RNA-seq_replicate_2.fq

Genome browser session
(e.g. UCSC)

http://genome-euro.ucsc.edu/s/VDR_group_public_data/Carrico_et_al_2020_ZebrafishPancreasRegulome

Methodology

Replicates	2 biological replicates from a pool of different zebrafish individuals of similar age (n>=10); replicate agreement: 0.78 Pearson's correlation coefficient.
Sequencing depth	Samples were sequenced on Illumina HiSeq 2000 platform, 49bp, single end.<br>Whole pancreas H3K27ac ChIP-seq replicate 1: 19453108 total reads, 12445065 (63.97%) uniquely mapped reads<br>Whole pancreas H3K27ac ChIP-seq replicate 2: 27406818 total reads, 15425471 (56.28%) uniquely mapped reads
Antibodies	-rabbit anti-H3K27ac antibody (1:2, #ab4729, Abcam)<br>-rabbit anti-H3K4me3 antibody (1:5, #ab8580, Abcam)
Peak calling parameters	Bowtie2 (v2.6.6) was used for mapping against the zebrafish genome (GRCz10/danRer10) with default parameters, e.g. bowtie2 -x danRer10 -U chip1.fq -p 10 -S chip1.sam.<br>MACS (v1.4.2) was used for peak calling with the parameters "--nomodel, --nolambda and --space=30", after converting the alignment bam to bed file and sorting.
Data quality	Raw reads were of high quality (supplied fastqc html reports).<br>For replicate 1, there are 46107 peaks with p-value <= 0.05 & FC >= 5.<br>For replicate 2, 22494 peaks with p-value <= 0.05 & FC >= 5.<br>Replicate agreement: 0.78 Pearson's correlation coefficient.
Software	Read QC - FASTQC v.0.11.5; remove adapters - Skewer v.0.2.1; genome mapping - Bowtie2 v.2.2.6; file operations - Bedtools v.2.7.1; peak calling - MACS14 v.1.4.2; file validation - bedClip v.1.04.00, peak annotation and motif enrichment discovery - Homer v.4.11.1; file conversion and operations - samtools v.1.9; genomic coordinate transformation - liftOver v.1.04.00; genome coverage - genomeCoverageBed v.1.04.00; file conversion - bedGraphToBigWig v.1.04.00; read density heatmap - seqMiner v.1.3.4.

Flow Cytometry

Plots

Confirm that:

☒ The axis labels state the marker and fluorochrome used (e.g. CD4-FITC).

☒ The axis scales are clearly visible. Include numbers along axes only for bottom left plot of group (a 'group' is an analysis of identical markers).

☐ All plots are contour plots with outliers or pseudocolor plots.

☐ A numerical value for number of cells or percentage (with statistics) is provided.

Methodology

Sample preparation	The whole pancreases were dissected from double transgenic adult zebrafish [Tg(ins:GFP, ela:mCherry), Tg(ins:GFP, gcga:mCherry), and Tg(ins:GFP, sst:mCherry)] and fixed using 4% formaldehyde in 1xPBS. Cells were dissociated, on ice, using a 15 mL Dounce homogenizer in 1 mL of ice-cold sort buffer (1% EDTA, 2mM HEPES pH 7.0 in 1xPBS), and then passed through a 40-µm cell strainer. Immediately following dissociation, the mCherry and GFP fluorescence were analyzed on a BD FACS-ARIA II cell sorter (BD Biosciences) to assess the relative proportion of pancreatic cell types.
Instrument	FACS-ARIA II cell sorter (BD Biosciences).
Software	FlowJo 10.2 software.
Cell population abundance	Tg(ins:GFP, ela:mCherry) animals: acinar cells and beta-cells were 0.75±0.24% and 0.14±0.03% of the total population, respectively.<br>Tg(ins:GFP, gcga:mCherry) animals: beta-cells and alpha-cells were 2.31±1.84% and 0.28±0.23% of the total population, respectively.<br>Tg(ins:GFP, sst:mCherry) animals: beta-cells and delta-cells were 2.38±1.50% and 0.36±0.25% of the total population, respectively.
Gating strategy	For flow-cytometric analysis of the different pancreatic cell populations, cells were gated using standard methods for all experiments: 1) FSC/SSC gate to identify living cells, 2) FSC-H/FSC-A to identify single cells, and 3) single cells were gatted in relation to the control samples (pancreases from wild-type animals) according to positivity/negativity for reporter expression;<br>- Tg(ins:GFP, ela:mCherry) animals: The beta-cell population was defined by gating GFP Positive mCherry Negative single cells, and the acinar cell population was defined by gating GFP Negative mCherry Positive single cells,<br>- Tg(ins:GFP, gcga:mCherry) animals: The beta-cell population was defined by gating GFP Positive single cells, and the alpha-cell population was defined by gating GFP Negative mCherry Positive single cells,<br>- Tg(ins:GFP, sst:mCherry) animals: The beta-cell population was defined by gating GFP Positive mCherry Negative single cells, and the delta-cell population was defined by gating GFP Negative mCherry Positive single cells.

☐ Tick this box to confirm that a figure exemplifying the gating strategy is provided in the Supplementary Information.
